# Effects of Unconventional Supplementary Cementitious Materials on the Mechanical Strength and Environmental Benefits of Cement-Based Materials—Multi-Objective Optimization for Separately Modified Mortars

**DOI:** 10.3390/ma19214618

**Published:** 2026-10-30

**Authors:** Jiayuan Xu, Xu Zhan, Wei Wang, Ruisen Li

**Affiliations:** 1 Department of Information Resources Management, School of Economics and Management, University of Chinese Academy of Sciences, Beijing 100190, China; 2 National Science Library, Chinese Academy of Sciences, Beijing 100190, China; 3 Department of Civil Engineering, Architecture and Environment, Instituto Superior Técnico, Universidade de Lisboa, Av. Rovisco Pais 1, 1049-001 Lisbon, Portugal; 4 School of Civil Engineering, Harbin Institute of Technology, Harbin 150039, China; 5 College of Civil Engineering & Architecture, Qingdao Agricultural University, Qingdao 266109, China

**Keywords:** common waste, mechanical properties, economic and environmental analysis, artificial intelligence, multi-objective optimization

## Abstract

This study experimentally investigates the mechanical properties and development patterns of mortars incorporating steel slag (SS), coal gangue (CG), and biochar (BC) and establishes a multi-objective optimization framework for sustainable mix design. The investigation systematically evaluates the individual effects of these materials on mechanical properties, economic cost, and environmental impact, providing a comparative basis for single-SCM mix design. Experimental results demonstrate distinct material-specific behaviors: SS substitution (10–40%) exhibits behavior consistent with latent hydraulic activity, mitigating early-age strength loss and showing a relative 28-day strength recovery from 71.4% to 80.5% at 30% substitution. CG acts primarily as a filler-type component under the tested conditions, causing a progressive, dosage-dependent strength reduction, with 28-day relative strength declining to 46.4% at 40% substitution. In contrast, BC at low dosages (1–2%) functions as a performance-enhancing cement replacement, improving 28-day compressive strength by up to 11.7% within the tested curing period through physical effects supported by the previous literature. Preliminary environmental screening indicates that both SS and CG significantly reduce cost (down to 2421.6 and 2410.2 CNY/m^3^, respectively), energy consumption (down to 3645.3 and 3028.6 MJ/m^3^), and carbon footprint (down to 492.2 and 438.0 kg CO_2_-eq/m^3^) at 40% substitution. Conversely, BC increases cost and energy demand but reduces carbon emissions (619.8 kg CO_2_-eq/m^3^ at 4% replacement) under the adopted carbon sequestration assumption. The optimization model successfully identifies Pareto-optimal mixtures that balance the trade-offs between superior mechanical performance, cost-effectiveness, and reduced environmental impact, providing viable strategies for developing sustainable construction materials through the separate utilization of industrial and agricultural waste streams.

## 1. Introduction

The decarbonization of the construction industry has become a global imperative, with cement production alone contributing 8–10% of anthropogenic CO_2_ emissions—primarily from limestone calcination (CaCO_3_ → CaO + CO_2_) and fossil fuel combustion [1,2,3,4]. As urban infrastructure expands to support a projected population of 9.7 billion by 2050, annual cement demand is expected to exceed 5 gigatons, amplifying environmental pressures. To solve this issue, the utilization of supplementary cementitious materials (SCMs) in mortars to replace cement can reduce carbon emissions effectively [5,6,7]. Traditional SCMs such as fly ash and granulated blast furnace slag, though effective, face supply constraints due to the global phase-out of coal-fired power plants and shifts in steel manufacturing [8,9,10,11,12]. Consequently, the valorization of non-conventional industrial by-products, including steel slag, coal gangue, and biochar, has emerged as a critical pathway to reduce clinker consumption, divert waste from landfills, and enhance concrete durability, exhibiting great potential for relieving the environmental burden.

To be more specific, steel slag (SS) is a by-product of basic oxygen or electric arc furnaces, showing potential hydration properties owing to its mineralogical composition dominated by γ-dicalcium silicate (γ-C_2_S), tricalcium silicate (C_3_S), and iron oxides [13,14,15]. When the particle size of SS is finely ground to a certain extent, this material can participate in the hydration reaction to form hydration products, thus densifying the matrix [16]. In contrast, coal gangue (CG) is a kaolinite-rich residue from coal mining, which requires calcination at 700–800 °C to dehydroxylate its clay minerals into reactive metakaolin [17,18,19]. This activated phase consumes portlandite to generate secondary C-A-S-H, which shows beneficial effects on the long-term strength of cement-based materials and can further suppress alkali–silica reaction (ASR) expansion via alkali ion sequestration. Moreover, biochar (BC) produced from pyrolyzed biomass (350–700 °C) serves as a multifunctional SCM due to its hierarchical microporosity (300–800 m^2^/g specific surface area) [20,21,22]. BC can act as an internal curing agent to reduce the autogenous shrinkage of cement-based materials, and the oxygenated surface functional groups (-COOH and -OH) of BC further enable heavy metal immobilization [22,23].

Although the material-level behavior of SS, CG, and BC has been widely investigated, the prediction and optimization of sustainable cementitious mixtures have also attracted increasing attention. Previous studies have predominantly focused on assessing the fundamental feasibility of using SS, CG, and BC as SCMs to partially replace cement in mortar production. Some studies employed data-driven models such as ANN, RF, and XGBoost for strength prediction, while others applied life-cycle assessment to evaluate the environmental benefits of sustainable mixtures. However, most of these studies either examined a single type of SCM, focused primarily on prediction accuracy, or considered mechanical, economic, and environmental objectives in isolation. Compared with previous studies on biochar-modified cementitious composites, response-surface optimization, life-cycle assessment of sustainable concrete, and ANN-based performance prediction [24,25,26,27,28], the present work does not aim to increase the number of citations or to claim a fully comprehensive LCA. Its specific contribution is a consistent single-SCM comparison under the same water-to-binder ratio and curing conditions, combined with an explicit interpolation-based strength model and a multi-objective optimization framework that simultaneously considers strength, cost, energy consumption, and carbon emissions. Consequently, significant gaps remain. In particular, there is still a lack of quantitative and critical comparison of the precise and quantifiable effects of these SCMs on two key performance categories: mechanical strength development and environmental impact mitigation. This gap is especially pronounced in holistic, systematic research aimed at multi-objective optimization. There is a critical lack of integrated studies that simultaneously model, analyze, and optimize the complex interactions among the three core performance criteria: achieving required mechanical strength (performance), minimizing overall economic cost (economy), and maximizing genuine environmental benefits (sustainability). Since SS, CG, and BC exhibit different mechanical, economic, and environmental behaviors, they need to be investigated under the same water-to-binder ratio and curing conditions so that their performance can be compared on a consistent basis.

To address this issue and provide a sustainable mortar design strategy with considerable mechanical strength and promising economic and environmental benefits, this study innovatively conducted a consistent comparison of three unconventional SCMs under identical water-to-binder ratio and curing conditions, and established a single-SCM multi-objective optimization framework using explicit interpolation-based strength models. Specifically, the compressive strength development of mortars containing various amounts of steel slag (SS), coal gangue (CG), and biochar (BC) was systematically investigated, with replacement ratios of 0–40 wt% for SS and CG and 0–4 wt% for BC. In addition, the energy consumption and embodied CO_2_ emissions during mortar production were evaluated. Subsequently, a single-SCM multi-objective optimization framework was established using NSGA-III and DE algorithms to balance compressive strength, cost, energy consumption, and carbon emissions. This work provides new insights into the sustainable development of green mortars incorporating unconventional waste materials as SCMs.

## 2. Experimental Methods

### 2.1. Raw Materials

**Figure 1:**
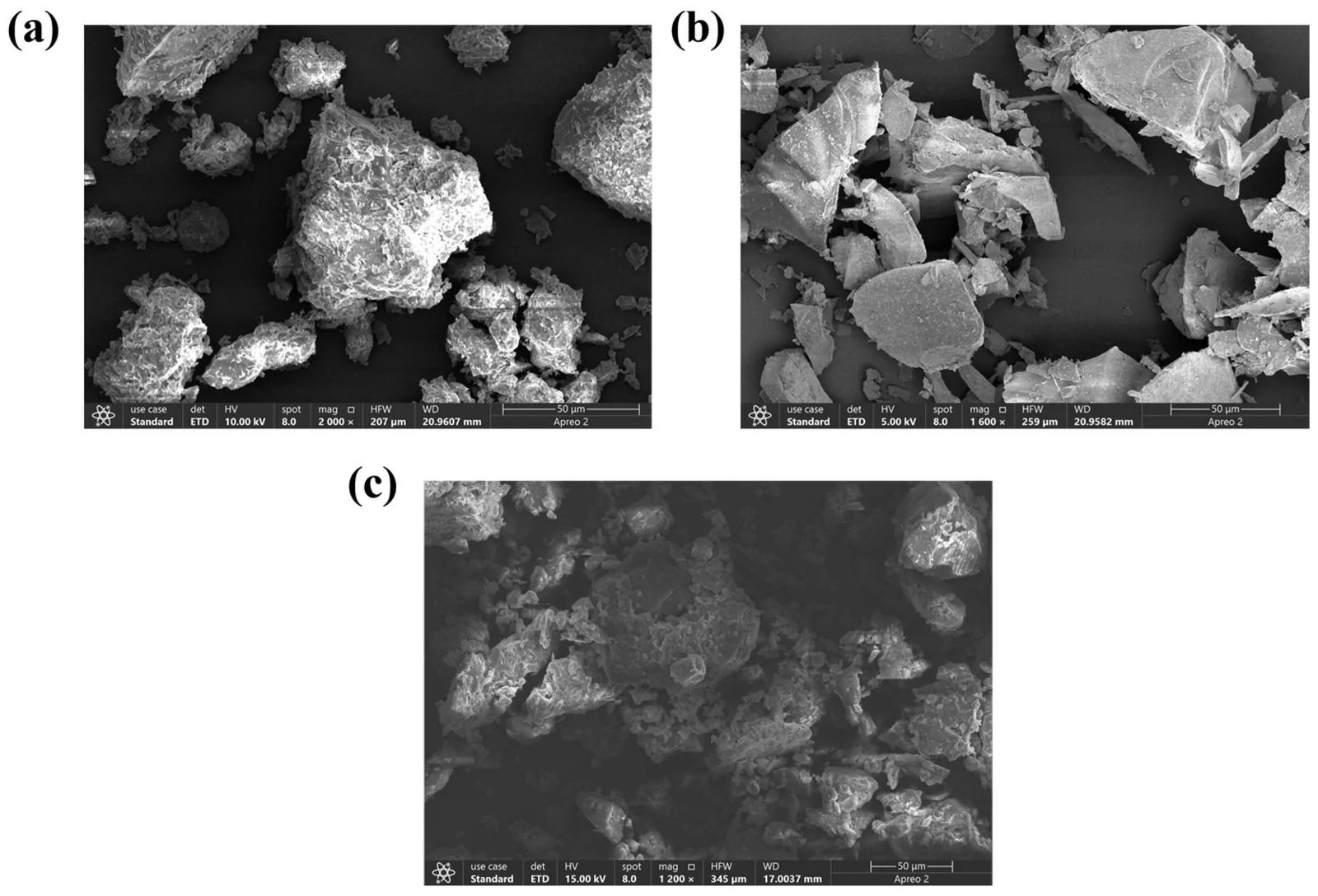
SEM images of cementitious powder materials: (**a**) SS, (**b**) CG and (**c**) BC.

Ordinary Portland cement (strength grade 42.5 MPa) was used as the main cementitious material and was supplied by Nanchang Conch Cement Co., Ltd., Nanchang, Jiangxi, China. Steel slag (SS) was supplied by Hebei Lingshou Dexu Mining Co., Ltd., Lingshou County, Shijiazhuang, Hebei, China. Coal gangue (CG) was supplied by Shijiazhuang Yuanjing Mineral Products Co., Ltd., Shijiazhuang, Hebei, China. Biochar (BC) was supplied by Wuxi Xingyuan Environmental Protection Technology Co., Ltd., Wuxi, Jiangsu, China. SS, CG, and BC were separately used as supplementary cementitious materials (SCMs) to replace cement. Fine aggregate with a particle size smaller than 2 mm was used and was supplied by Xiamen ISO Standard Sand Co., Ltd., Xiamen, Fujian, China. SS, CG, and BC were pretreated to have the same particle size range (0–75 μm) as cement. The chemical compositions of cement, SS, CG, and BC were determined by X-ray fluorescence spectrometry using a PANalytical Axios X-ray fluorescence spectrometer (Malvern Panalytical B.V., Almelo, The Netherlands). The chemical compositions are listed in Table 1, and the SEM images are presented in Figure 1. SEM imaging was performed using a Hitachi Regulus8100 scanning electron microscope (Hitachi High-Tech Corporation, Tokyo, Japan).

### 2.2. Mix Proportions and Preparation Method

In this study, SS, CG, and BC were used as cement replacements by mass. The replacement ratio was defined as the mass percentage of SCM relative to the total binder mass. Table 2 lists the mix proportions designed for the unconventional SCM blended mortars in this work. The water-to-binder ratio was kept constant at 0.35, and the same superplasticizer dosage was used for all mixtures to maintain comparable workability. Quantitative flow spread was not measured in this study. It should be emphasized that, as is common in concrete production, the replacement of cement with BC was kept lower than 5% to ensure good performance, so the BC contents were different from those of the SS and CG blended specimens. The preparation method is detailed below: All of the dry raw materials were stirred in a mixer for 3 min, and water was added, followed by a further 4 min of stirring, after which the fresh mixture was put into plastic molds and vibrated for 3 min to remove bubbles. Then, the specimens were cured at 20 ± 2 °C with a relative humidity >95%, and the curing ages of the specimens were set at 3 days, 7 days and 28 days; the mechanical strengths were tested at each of the curing ages.

### 2.3. Mechanical Strength Test

The compressive strengths of the specimens were measured according to GB/T 17671-2021 [29] at the curing ages of 3 days, 7 days and 28 days. During the test process, the test velocity was set at 2.4 kN/s. The specimen geometry was 40 mm × 40 mm × 40 mm. For each mixture and each curing age, three replicate specimens were tested. The compressive strength was calculated as the arithmetic mean of the three specimens. The standard deviation and coefficient of variation were calculated. The reported replication is specimen-level replication within one batch, not independent batch-level replication. One-way analysis of variance followed by Tukey HSD testing was used to compare replacement levels at each curing age where the data supported such a comparison. Differences were considered significant at *p* < 0.05. Because batch-level replication was not performed, relatively modest strength improvements are discussed as indicative rather than definitive.

## 3. Results and Discussion

### 3.1. Compressive Strength Development

**Figure 2:**
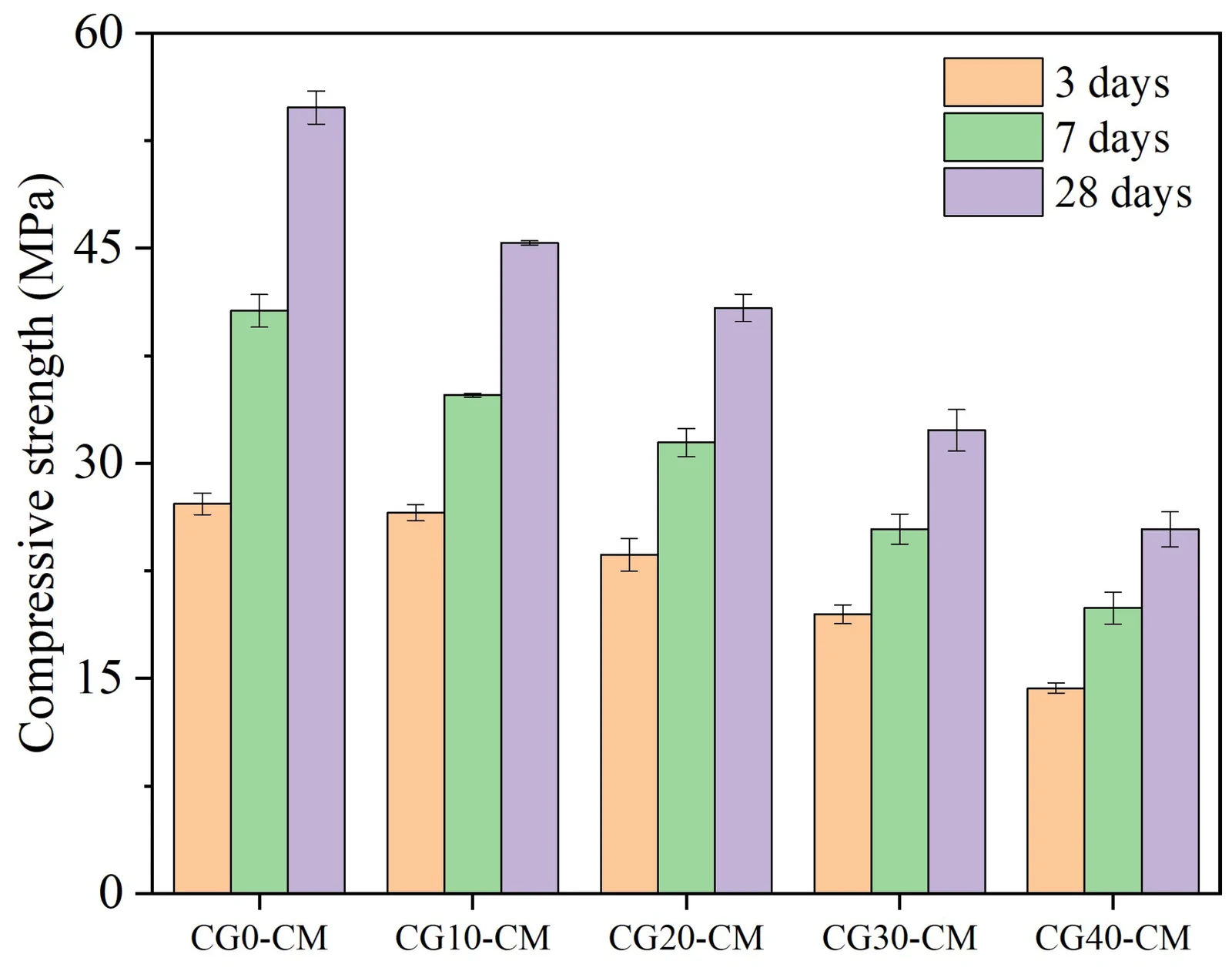
Compressive strengths of CG-CM at different ages.

Figure 2 depicts the compressive strengths of CG-CM specimens. It can be found that the incorporation of CG progressively reduces the compressive strength at different ages. Moreover, this reduction exhibits a distinct dosage-dependent relationship, where higher CG substitution levels contribute to more significant strength decreases. To be specific, the 3-day compressive strength experienced a gradual decline from 27.17 MPa for CG0-CM to 14.30 MPa for CG40-CM, representing a 47.4% reduction at the highest substitution level. This early-age strength reduction is interpreted, with support from the previous literature, as primarily reflecting a filler-type dilution effect during the initial hydration period [30]. Moreover, the 28-day compressive strength, representing the 28-day performance within the tested period, decreased from 54.80 MPa to 25.40 MPa with increasing CG contents. Notably, the absolute strength reduction becomes more pronounced at later ages, which may indicate that the pozzolanic activity of CG insufficiently compensates for the cement dilution effect throughout the prolonged curing period.

**Figure 3:**
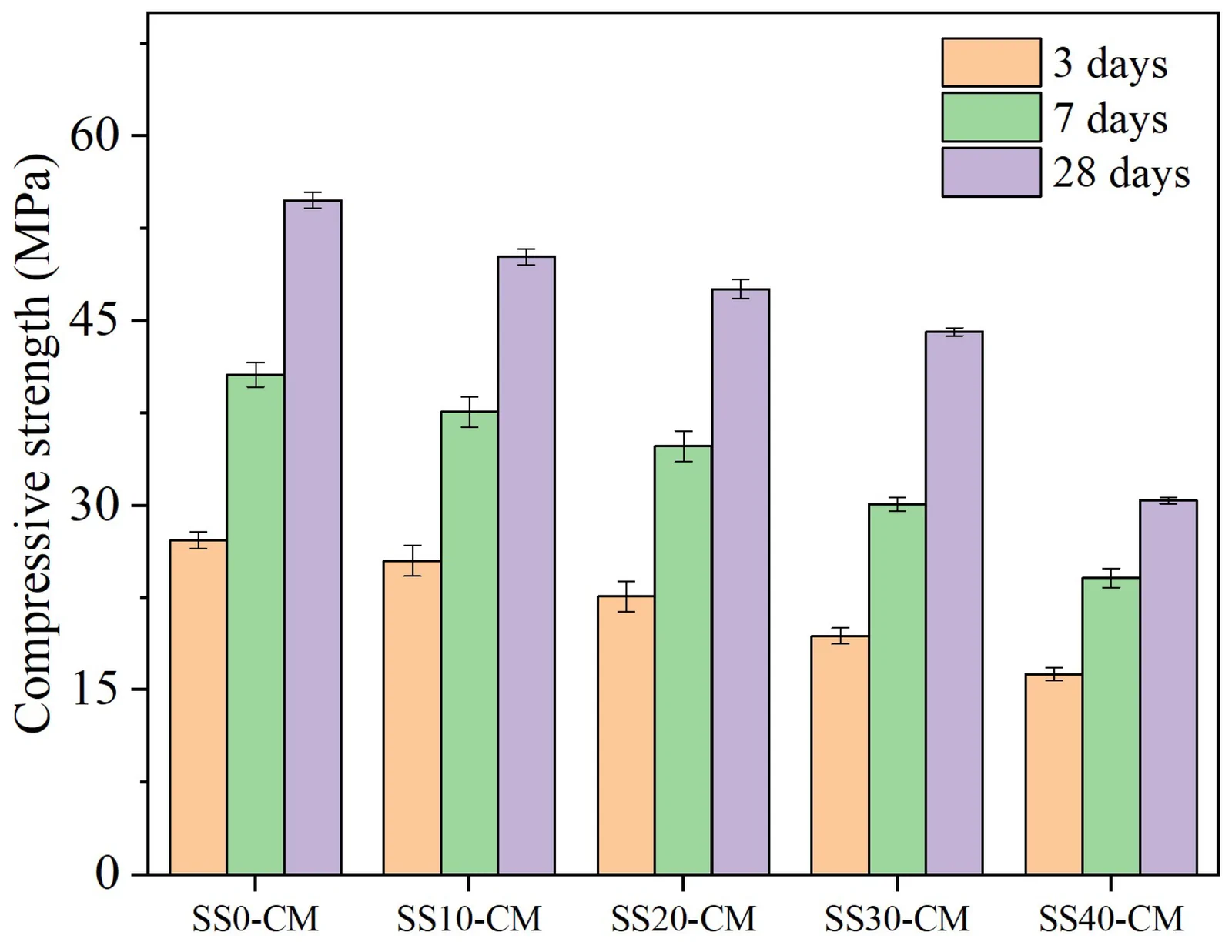
Compressive strengths of SS-CM at different ages.

The compressive strengths of SS-CM are summarized in Figure 3. The results revealed a distinct influence of SS substitution on mechanical performance throughout the curing period; a systematic reduction in compressive strength with increasing SS content was found across all curing ages. However, the magnitude of this reduction exhibited notable variations depending on both the substitution rate and the curing duration. Specifically, at early ages, the compressive strengths decreased from 27.17 MPa to 16.30 MPa for SS40-CM, representing a 40% strength reduction. This early-strength decline can also be attributed to the dilution effect of SS on cement. Further, although the 28-day compressive strength still decreased as SS content increased, the retention rates at moderate substitution levels (10–30%) were notably higher than those observed for CG at equivalent substitution rates. For instance, SS30-CM maintained 80.5% of the control group’s 28-day strength, compared to approximately 58.9% for the CG30-CM. This improved performance is interpreted, with support from the previous literature, as behavior consistent with possible pozzolanic or latent hydraulic properties of SS that contribute to strength development at later ages, partially compensating for the dilution effect [31,32].

**Figure 4:**
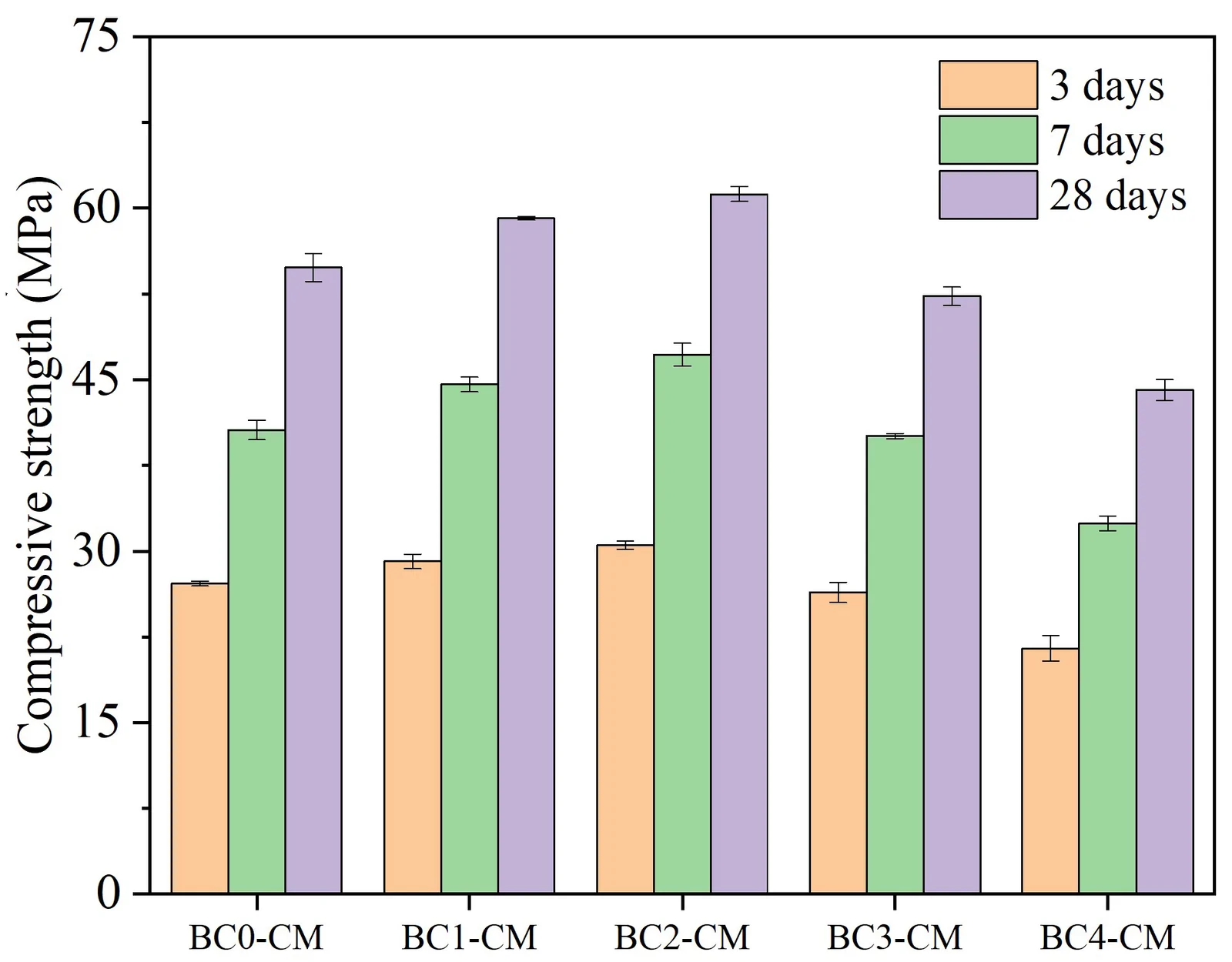
Compressive strengths of BC-CM at different ages.

The compressive strength development of BC-CM is illustrated in Figure 4. The results reveal a significantly different pattern compared to both CG and SS blended specimens. The experimental data demonstrate a strength enhancement at lower BC replacement levels followed by a reduction at higher contents. At the optimal dosage (2% BC replacement), significant strength improvements can be observed across all curing ages. To be precise, the 3-day compressive strength was increased by 12.3% compared to the control specimen, rising from 27.17 MPa to 30.50 MPa. This early-age enhancement is interpreted, with support from the previous literature, as possibly arising from nucleation effects and accelerated early hydration, rather than a mechanism directly demonstrated by the present experiments. The strength improvement became more pronounced at 7 days, with BC2-CM exhibiting a 16.2% increase relative to the control group (47.20 MPa versus 40.62 MPa). This consistent enhancement pattern may indicate that BC’s positive effects extend beyond initial hydration stages, possibly through internal curing mechanisms in which the porous structure of biochar gradually releases absorbed water to support continued hydration. The 28-day compressive strength reached 61.20 MPa for BC2-CM, representing an 11.7% improvement over the control specimen. This enhancement within the tested curing period suggests that appropriate BC incorporation may improve the performance of cement-based materials [33,34].

### 3.2. Relative Compressive Strength

**Figure 5:**
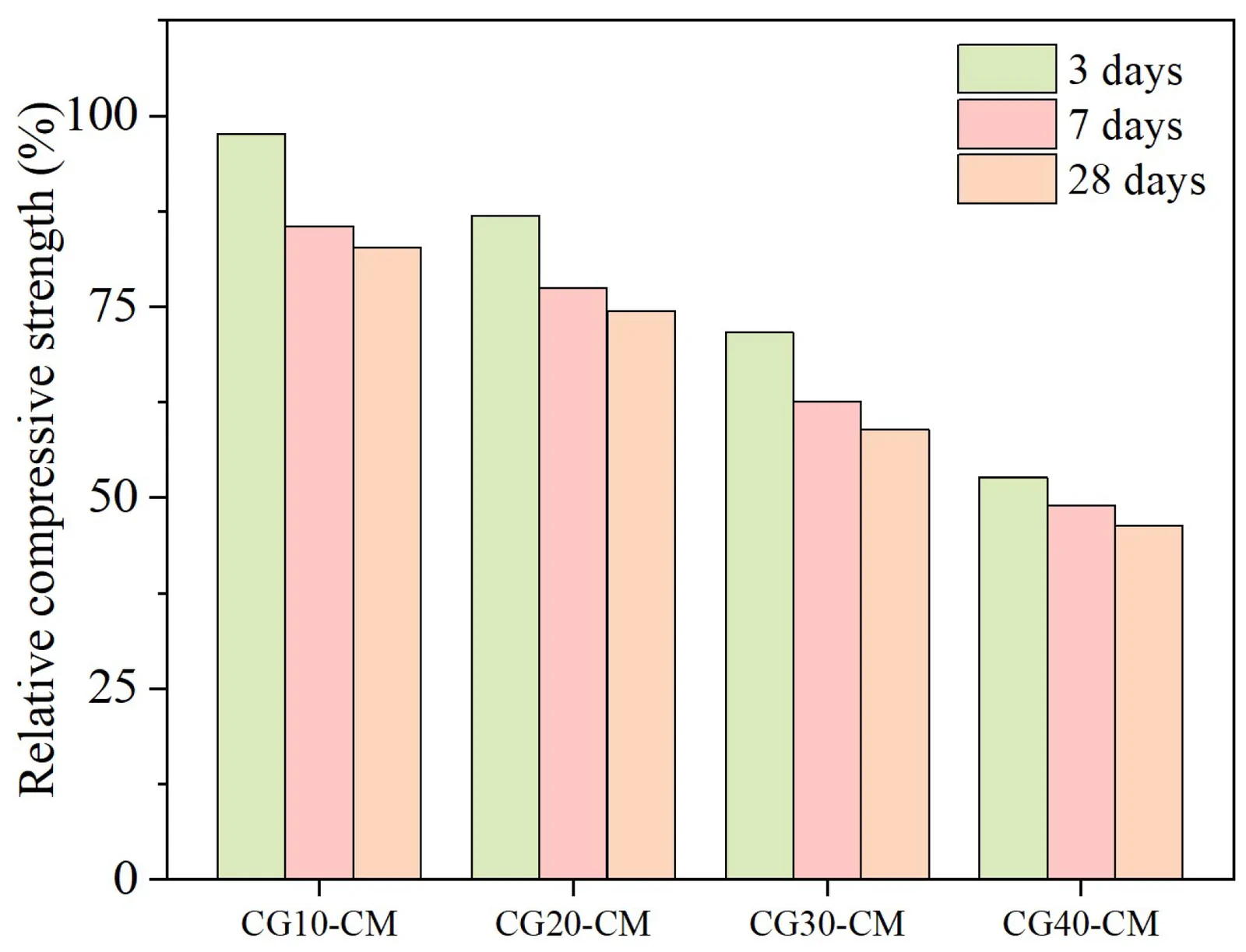
Relative compressive strength of CG-CM at different curing ages.

**Figure 6:**
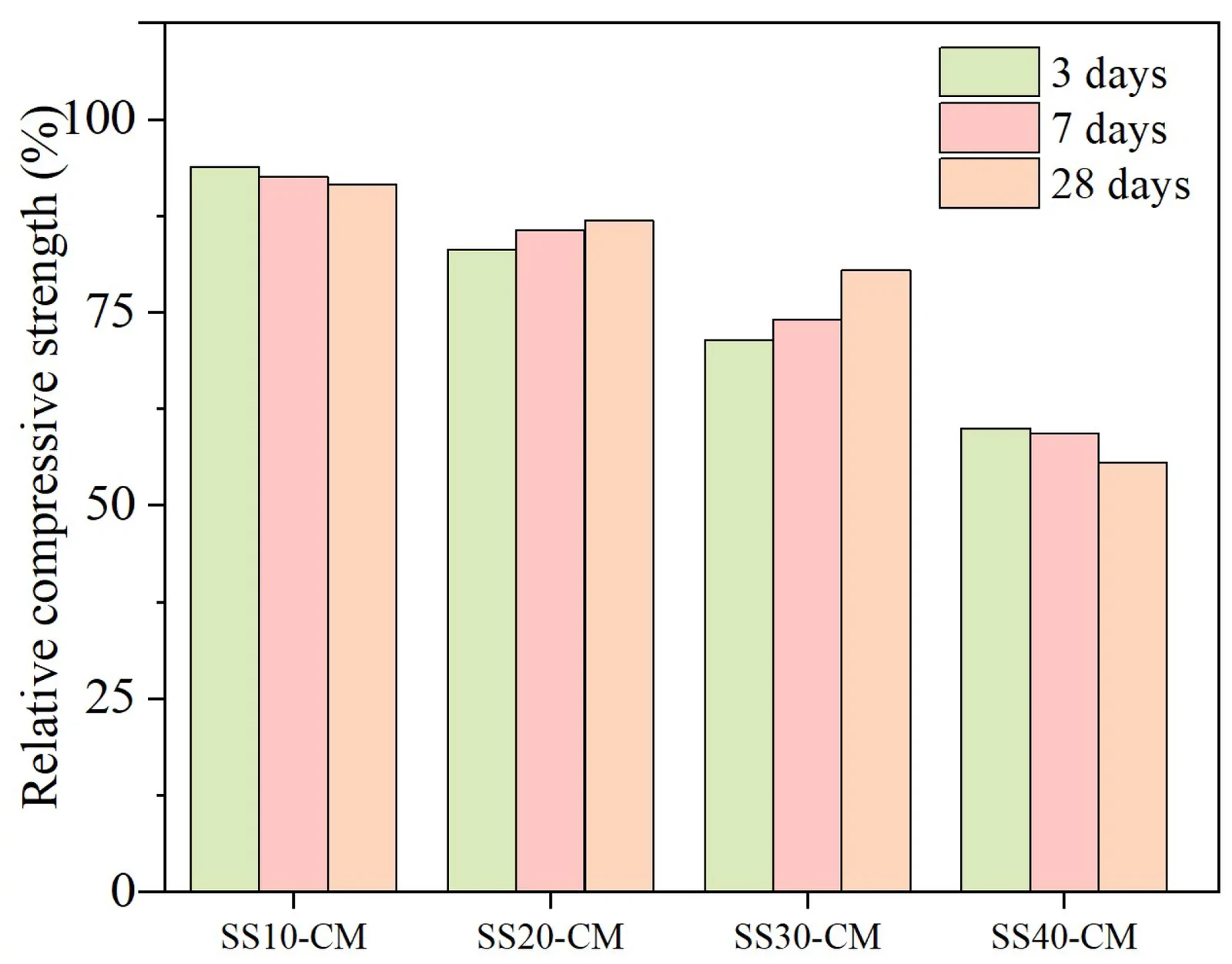
Relative compressive strength of SS-CM at different curing ages.

**Figure 7:**
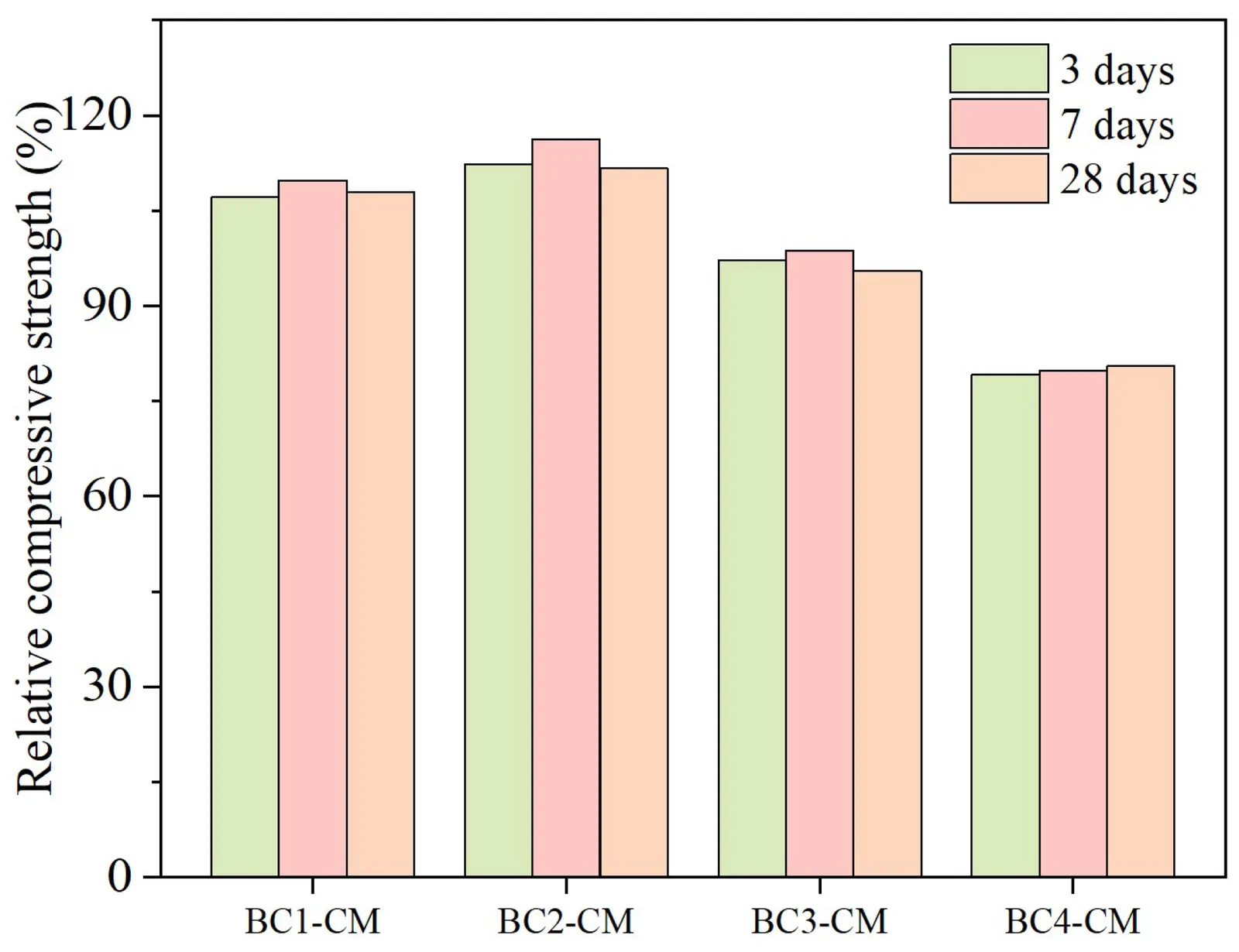
Relative compressive strength of BC-CM at different curing ages.

Figure 5, Figure 6 and Figure 7 systematically depict the relative compressive strength of the specimens containing various SCMs at different ages. For CG blended specimens, it can be found that the CG contents show a typical negative effect on the strength development across all curing ages. At the early curing age of 3 days, the detrimental effect of CG substitution was already evident. The relative compressive strength of CG10-CM was 97.7%, indicating a marginal but immediate strength loss due to the initial dilution of cement. This early strength reduction became more pronounced with increasing CG content, with the strength declining to 86.9% for CG20-CM, 71.6% for CG30-CM, and only 52.6% for CG40-CM. Similarly, this declining trend was significant at the longer curing age: the 28-day relative compressive strength decreased from 82.8% for CG10-CM to 74.5% for CG20-CM, 58.9% for CG30-CM, and 46.4% for CG40-CM. This consistent decline, coupled with a trend of lower relative strength at later ages for each individual specimen, is consistent with a primarily filler-type behavior of CG under the tested conditions.

In contrast, SS blended specimens exhibited a development trend indicative of potential hydration reactivity. To be specific, at a 10% substitution rate, the relative compressive strength remained high and stable, from 93.8% at 3 days to 91.6% at 28 days. More notably, at moderate substitution levels, the relative compressive strength demonstrated a clear recovery over time. For SS20-CM, it increased from 83.1% at 3 days to 86.9% at 28 days, and for SS30-CM a more pronounced recovery from 71.4% to 80.5% was observed. This trend is interpreted, with support from the previous literature, as possibly reflecting the slow hydration of SS that partially compensated for the initial dilution effect and mitigated strength loss at later ages. However, at 40% substitution, this compensatory mechanism can be overwhelmed, resulting in a lower relative strength that declined from 60.0% to 55.5%.

The inclusion of a low BC content can effectively improve the compressive strength of the specimen and exhibit a non-monotonic influence. Specifically, the relative compressive strength of BC1-CM ranged from 107.1% (at 3 days) to 107.8% (at 28 days), while BC2-CM showed even greater enhancement, from 112.3% to 111.7%, with a peak of 116.2% at 7 days. This suggests that BC may act as a nucleation site, potentially improving curing efficiency. However, a sharp transition occurred beyond the 2% substitution content. The relative compressive strength of BC3-CM fell to levels at or below the control (97.2% to 95.4%), and BC4-CM exhibited a substantially compromised performance, stabilizing around 80% at all ages. This indicates a narrow optimal dosage range, where the positive physical effects are maximized before negative impacts, such as pore formation or agglomeration, become dominant. The distinct trends of the three materials suggest their respective roles in the strength development of cement-based materials, and these identified roles provide the fundamental mechanical basis for the multi-objective optimization of mortars.

### 3.3. Economic and Environment Benefit Analysis

To provide a preliminary environmental screening, Table 3 lists the cost (CNY/kg), embodied energy (MJ/kg) and embodied CO_2_ of each component in cement-based materials (including cement, sand, water, PCE-SP, SS, CG and BC). The data presented in this study were obtained from literature surveys [35]. The environmental assessment follows a cradle-to-gate system boundary, including raw material acquisition, processing, and transportation. Steel slag inventory data are based on LCA studies from the World Steel Association. Coal gangue data are from the China Coal Industry Association, considering mining and stockpiling stages. Biochar data are based on the IPCC biochar guidelines, assuming agricultural waste as feedstock. The biogenic carbon in biochar is accounted for following the IPCC approach, where carbon stored in biochar is treated as sequestered. The value of −1 kg CO_2_/kg is derived from the assumed stable carbon fraction, the pyrolysis carbon yield, and the avoided decay of agricultural waste, and it is treated as a scenario assumption rather than a universal constant. Transportation distances are assumed to be 50 km for sand, 100 km for cement, 50 km for SS, 100 km for CG, and 200 km for BC, all carried by diesel truck. The inventory year is set to 2020–2024, and the geographical background is the Chinese grid. Allocation follows mass allocation for industrial by-products. A sensitivity analysis is performed for transportation distance, biochar carbon sequestration factor, and energy mix, and the resulting changes in carbon footprint are discussed as scenario-dependent.

To provide a preliminary environmental screening, Table 3 lists the cost (CNY/kg), embodied energy (MJ/kg) and embodied CO_2_ of each component in cement-based materials (including cement, sand, water, PCE-SP, SS, CG and BC). It can be found that the use of SS and CG and effectively lower the cost, embodied energy and embodied CO_2_ compared to cement. Although BC has a higher price and embodied energy than cement, its embodied CO_2_ is much lower than that of cement, with a remarkable negative carbon emission characteristic in the embodied CO_2_ value of −1 kgCO_2_/kg. The linear weighting formulae for cost, energy consumption and carbon emissions are as follows:

$$C=\sum m_{i}c_{i},E=\sum m_{i}e_{i},G=\sum m_{i}g_{i}$$

where $m_{i}$ is the mass of each component in 1 m^3^ and $c_{i},e_{i,}{\;andg}_{i}$ are from Table 3.

Table 4, Table 5 and Table 6 list the mass of each raw material required to produce 1 m^3^ concrete. It should be noted that the 1 m^3^ quantities in Table 4, Table 5 and Table 6 were converted from the laboratory-mix proportions. The conversion procedure is described as follows. The laboratory-batch masses were first summed for each mixture. The measured hardened density of the 40 mm × 40 mm × 40 mm specimens was then used to obtain the mass corresponding to 1 m^3^. Because the water-to-binder ratio and the binder-to-sand ratio were fixed, and because the SCM replaced cement on a mass-for-mass basis, the relative proportions in Table 4, Table 5 and Table 6 remain unchanged after normalization. The measured density was used as a consistency check rather than as a recalculation of the mass-for-mass replacement. A worked calculation is provided for SS30-CM. The laboratory mixture contained 440.51 kg cement, 188.79 kg SS, 1887.9 kg sand, 220.3 kg water, and 12.6 kg PCE-SP. The total mass was 2749.99 kg. Using the measured hardened density (ρh), the 1 m^3^ batch mass is ρh × 1 m^3^, and the scaling factor is ρh × 1 m^3^ divided by 2749.99 kg. The values in Table 5 are obtained by applying this scaling factor to each component. The same procedure applies to Table 4 and Table 6. Fresh density, yield, and absolute-volume checks are recommended for future mix-design studies.

**Figure 8:**
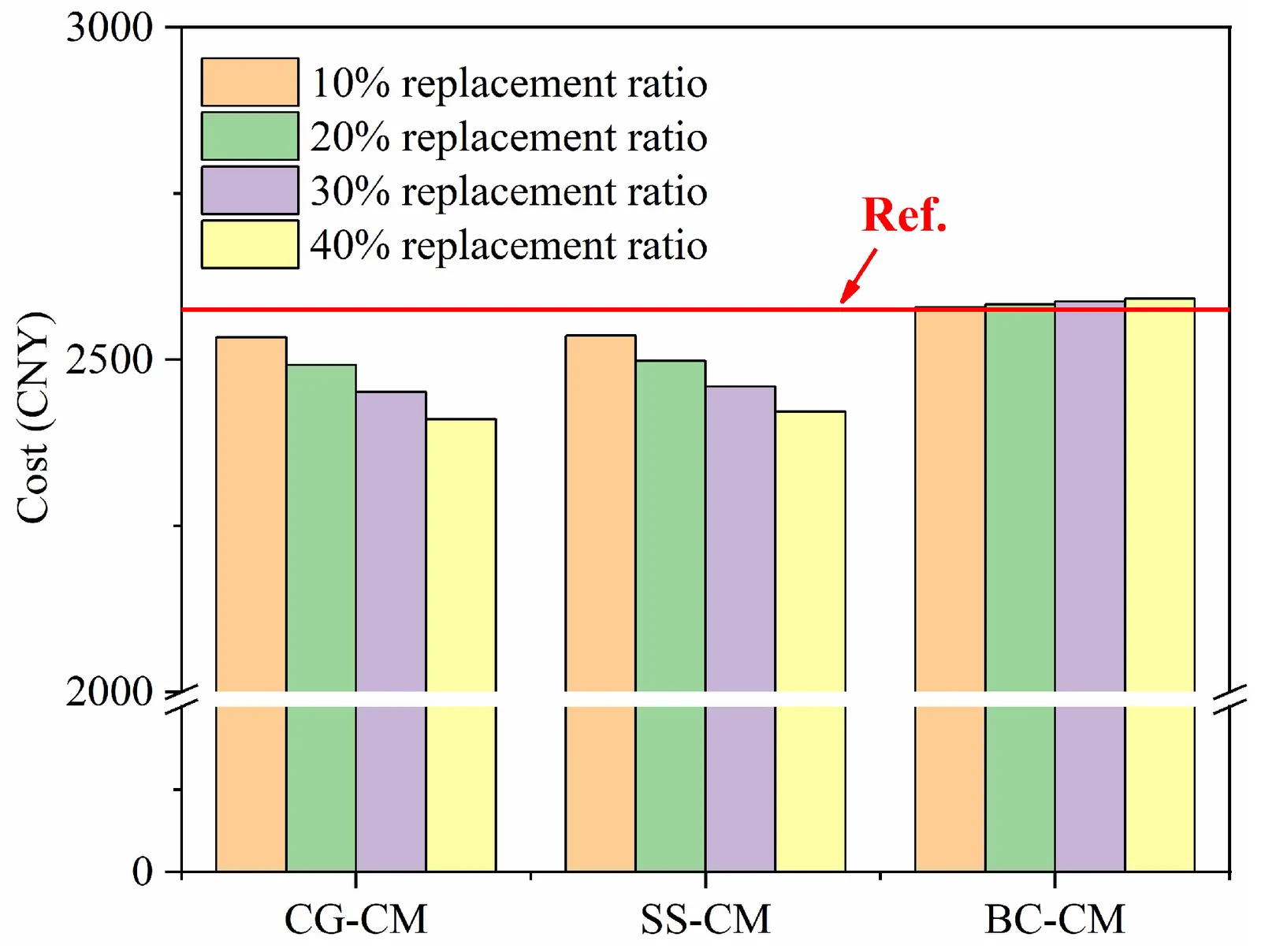
Total cost to produce 1 m^3^ concrete with different SCM substitution rates.

**Figure 9:**
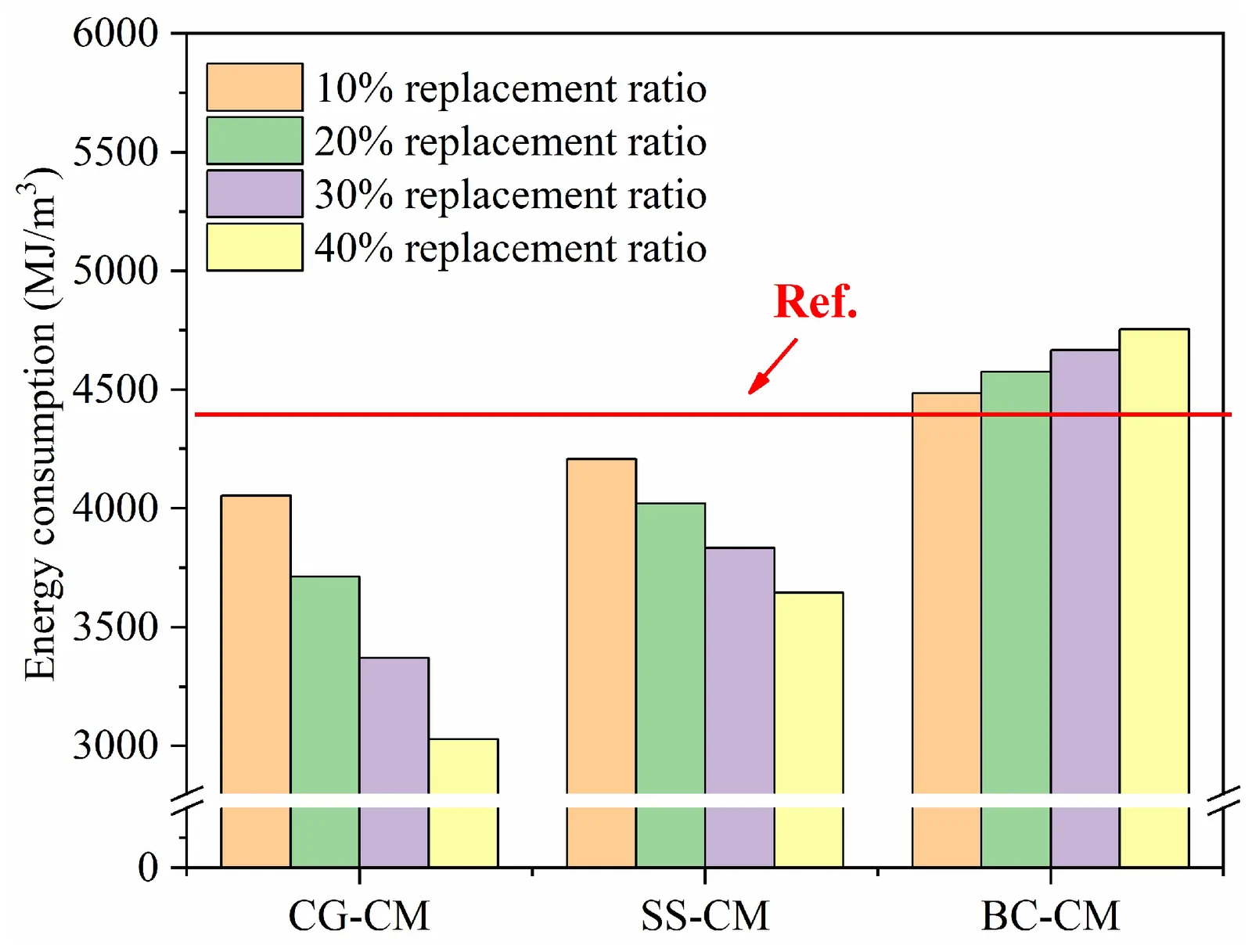
Total energy consumption to produce 1 m^3^ concrete with different SCM substitution rates.

**Figure 10:**
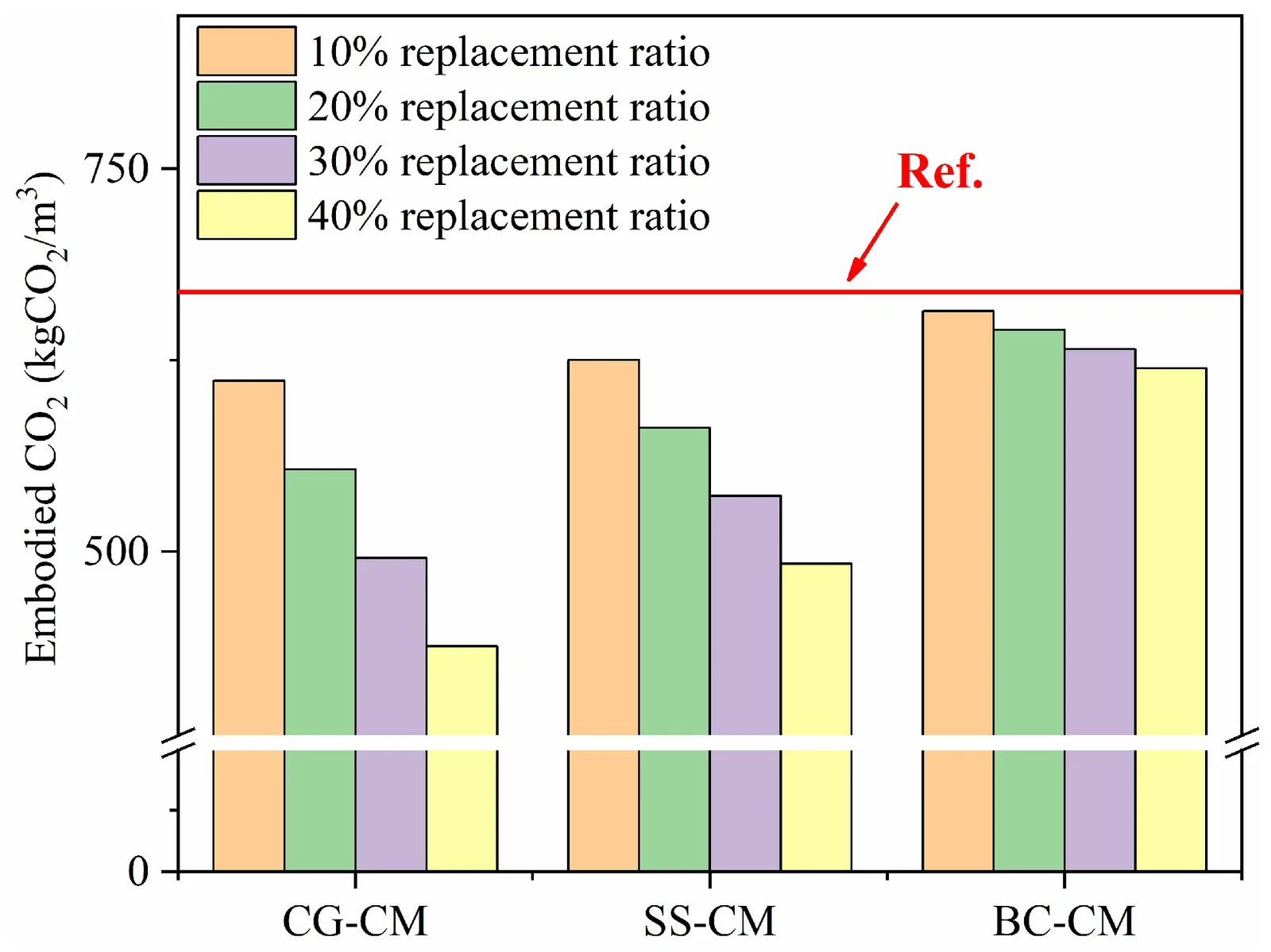
Embodied CO_2_ to produce 1 m^3^ concrete with different SCM substitution rates.

As shown in Figure 8, Figure 9 and Figure 10, the incorporation of both CG and SS can consistently reduce energy consumption and carbon emissions compared to the control group, with CG demonstrating a more pronounced efficiency. To be specific, at a 40% substitution rate, SS reduced the energy consumption and carbon footprint to 3645.3 MJ/m^3^ and 492.2 kg CO_2_-eq/m^3^, respectively, while CG achieves even lower values of 3028.6 MJ/m^3^ and 438.0 kg CO_2_-eq/m^3^, underscoring its superior environmental benefits stemming from the avoidance of clinker production and related calcination processes. Conversely, the use of BC led to a systematic increase in both cost and energy demand, with values rising from the baseline of 2574.6 CNY and 4396.0 MJ/m^3^ for the control to 2592.0 CNY and 4755.0 MJ/m^3^ at a 4% replacement rate, attributable to the energy-intensive pyrolysis production process of biochar; however, its carbon footprint shows a counteracting decrease to 619.8 kg CO_2_-eq/m^3^, suggesting that the carbon sequestration potential of BC partially offsets its embodied energy. From a cost perspective, both SS and CG contributed to a lower material cost, decreasing to 2421.6 CNY and 2410.2 CNY at 40% substitution, respectively, whereas BC induced a slight cost increase. These quantitative environmental and economic profiles, when integrated with the previously established mechanical performance data, can create a multi-dimensional objective space essential for formulating sustainable mortars that strategically balance mechanical performance, economic viability, and environmental impact.

To complement the 1 m^3^ functional unit, strength-normalized environmental indicators are also considered. For each mixture, cost, embodied energy, and embodied CO_2_ are divided by the 28-day compressive strength. This performance-normalized view helps to show whether a high-replacement mixture remains environmentally favorable when its lower mechanical performance is taken into account. For example, SS40-CM has a 28-day strength of 30.4 MPa and an embodied CO_2_ content of 492.2 kg CO_2_-eq/m^3^, corresponding to about 16.2 kg CO_2_-eq/m^3^ per MPa. CG40-CM has a 28-day strength of 25.4 MPa and an embodied CO_2_ content of 438.0 kg CO_2_-eq/m^3^, corresponding to about 17.2 kg CO_2_-eq/m^3^ per MPa. BC2-CM has a 28-day strength of 61.2 MPa and a lower strength-normalized carbon footprint than the control. Therefore, the apparent environmental ranking can change when mechanical performance is considered, and the strength-normalized indicators provide a more application-relevant basis for comparing mixtures with different replacement levels.

### 3.4. Multi-Objective Optimization

#### 3.4.1. Strength Model Establishment

**Figure 11:**
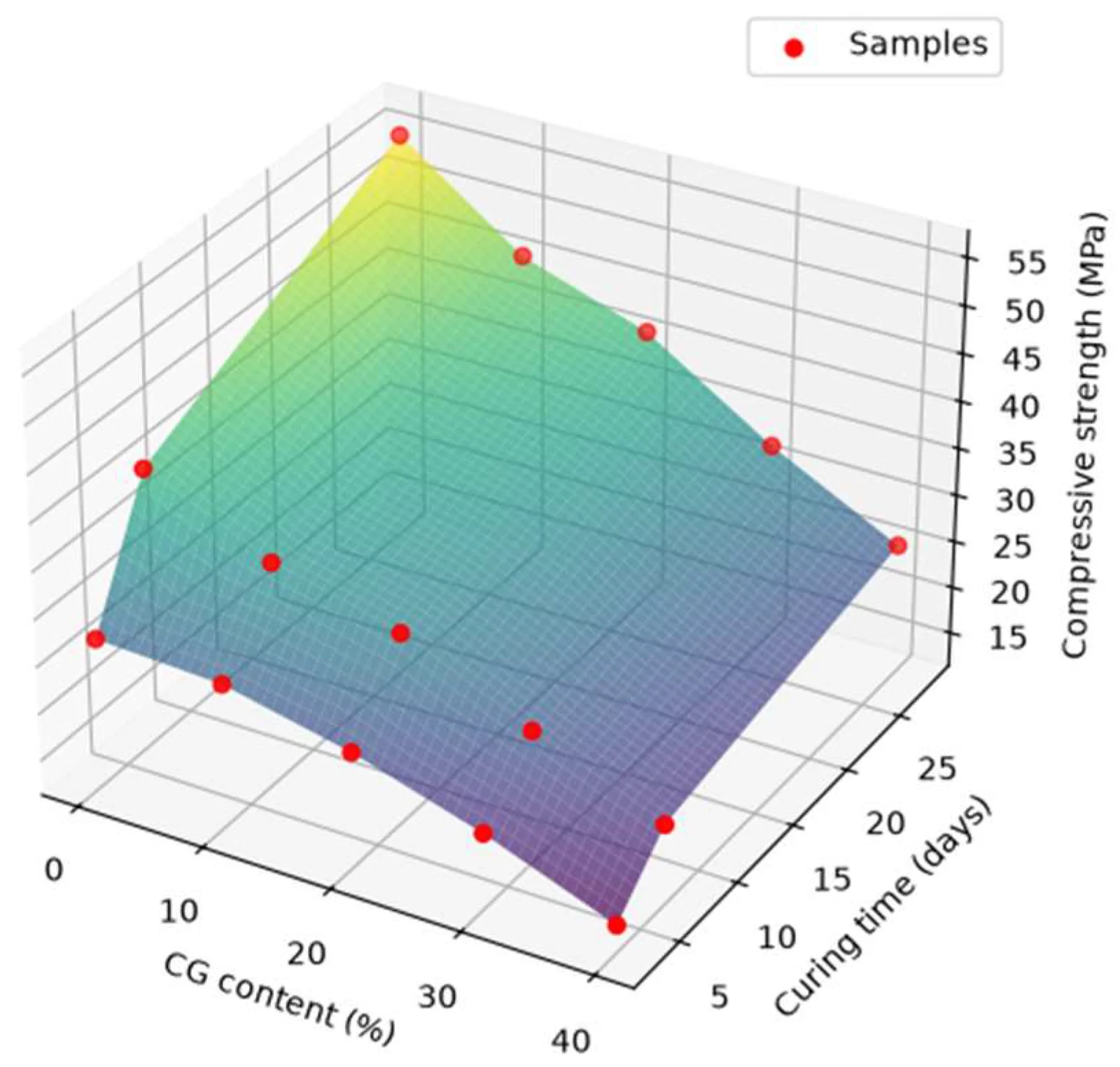
Model establishment of the relationship between CG content, curing time and compressive strength.

In this study, the multi-objective optimization was performed for each SCM separately to establish a single-material baseline. The interaction effects among SS, CG, and BC were not evaluated. A multivariate linear interpolation model was established to correlate CG content and curing time with compressive strength, as depicted in Figure 11, enabling strength prediction based on these two parameters. The performance of the multivariate linear interpolation method in this context was evaluated using Leave-One-Out Cross-Validation (LOOCV), yielding a mean MAE of 2.11. The standard deviation of the MAE across all LOOCV folds is 2.39 MPa. For CG, the dataset contains 15 observations. Eleven folds were included, and four boundary folds were excluded, namely, (0, 3), (0, 28), (40, 3), and (40, 28). The exclusion of boundary folds can produce an optimistic estimate of interpolation performance, so the reported MAE is regarded as a lower-bound estimate of prediction error. A simple multiple linear regression baseline was also evaluated, and the interpolation model provided a lower MAE than the baseline in the interior of the design space. The effect of prediction error on the subsequent Pareto solutions is discussed in the optimization section.

Explanation of LOOCV: For a dataset containing N samples, each sample is sequentially left out as the test set, while the remaining N − 1 samples are used to construct the interpolation model. The omitted sample is then used to evaluate the prediction accuracy. This process is repeated N times, ensuring that each sample is used exactly once as the test set. The average of the N-time evaluation results (in this study, N-time MAE values) serves as an estimate of the model performance. LOOCV is particularly suitable for scenarios with limited data.

**Figure 12:**
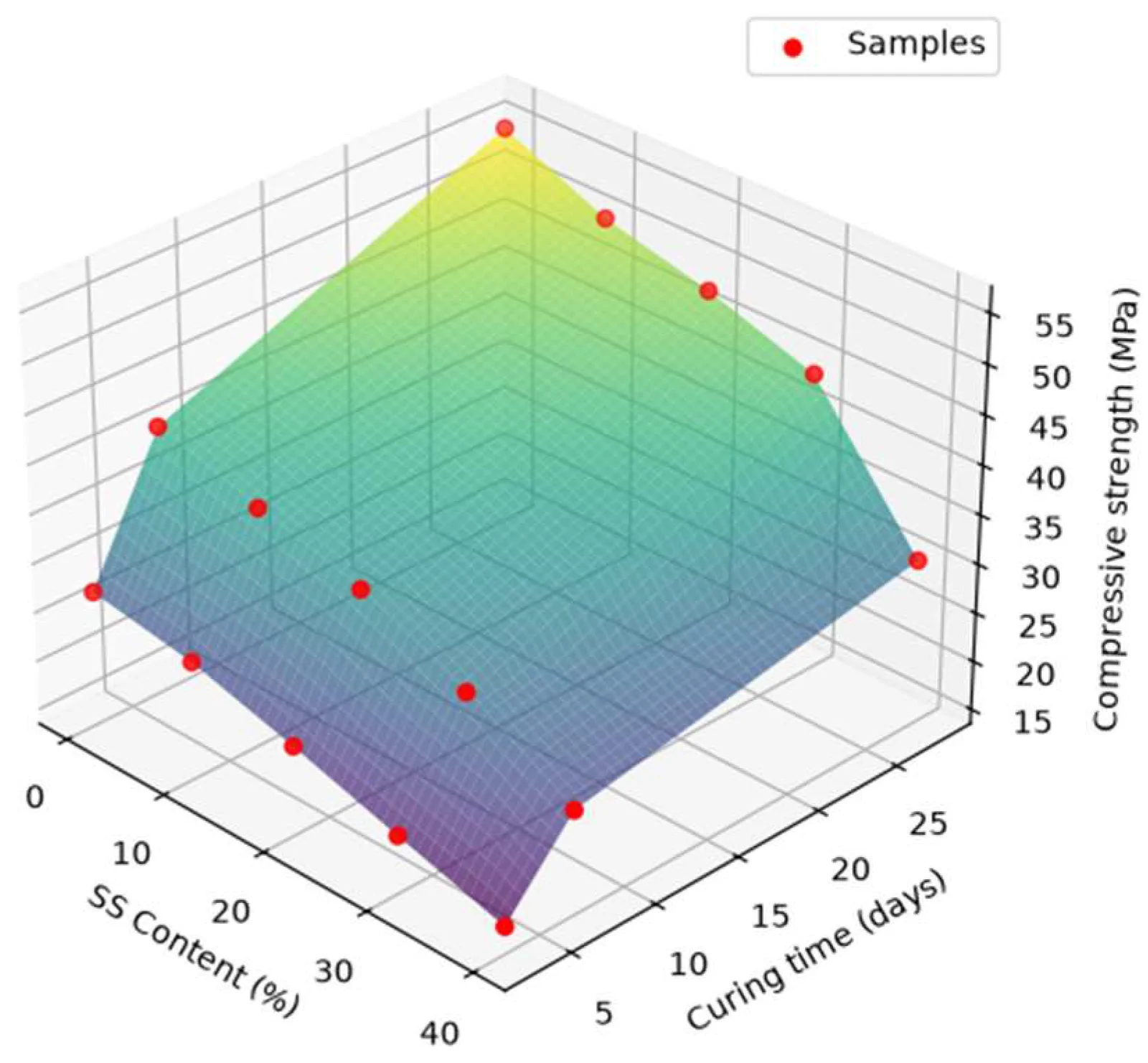
Model establishment of the relationship between SS content, curing time and compressive strength.

Moreover, a multivariate linear interpolation model was developed to describe the relationship between SS content, curing time, and compressive strength, allowing strength prediction based on these variables. The visualized interpolation surface, exhibited in Figure 12, indicated a good representation of the data, and the LOOCV evaluation resulted in a mean MAE of 2.15. The standard deviation of the MAE across all LOOCV folds is 2.87 MPa. For SS, the same four boundary folds were excluded, namely, (0, 3), (0, 28), (40, 3), and (40, 28).

**Figure 13:**
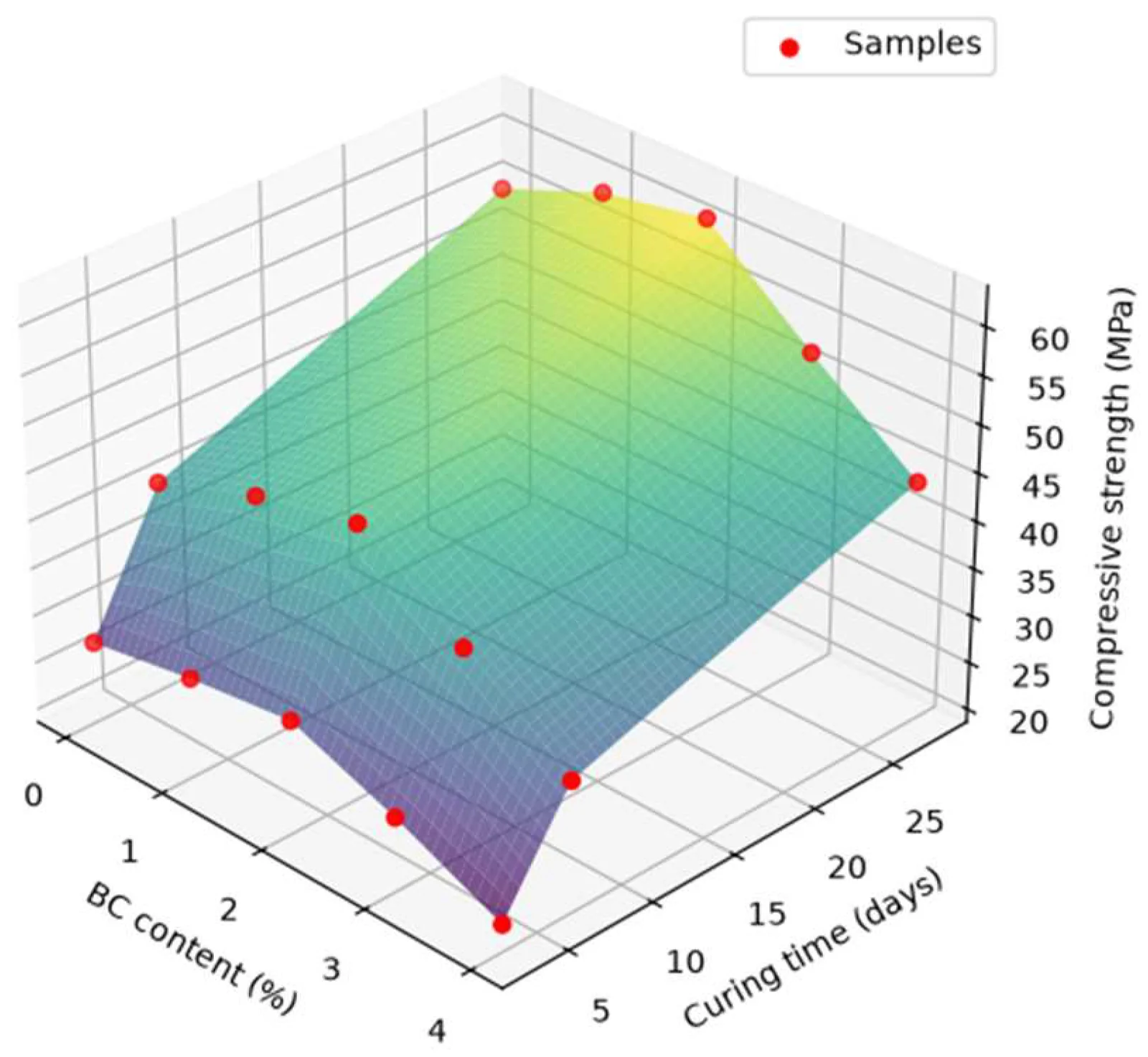
Model establishment of the relationship between BC content, curing time and compressive strength.

Further, a multivariate linear interpolation model was constructed to relate BC content and curing time to compressive strength, facilitating strength prediction using these parameters. The visualized interpolation surface also showed a reasonable fit, as illustrated in Figure 13, as the LOOCV evaluation produced a mean MAE of 2.96. The standard deviation of the MAE across all LOOCV folds is 3.04 MPa. For BC, the excluded boundary folds are (0, 3), (0, 28), (4, 3), and (4, 28).

#### 3.4.2. Bi-Objective Analysis and Pareto Front Solutions

##### Bi-Objective Analysis

**Figure 14:**
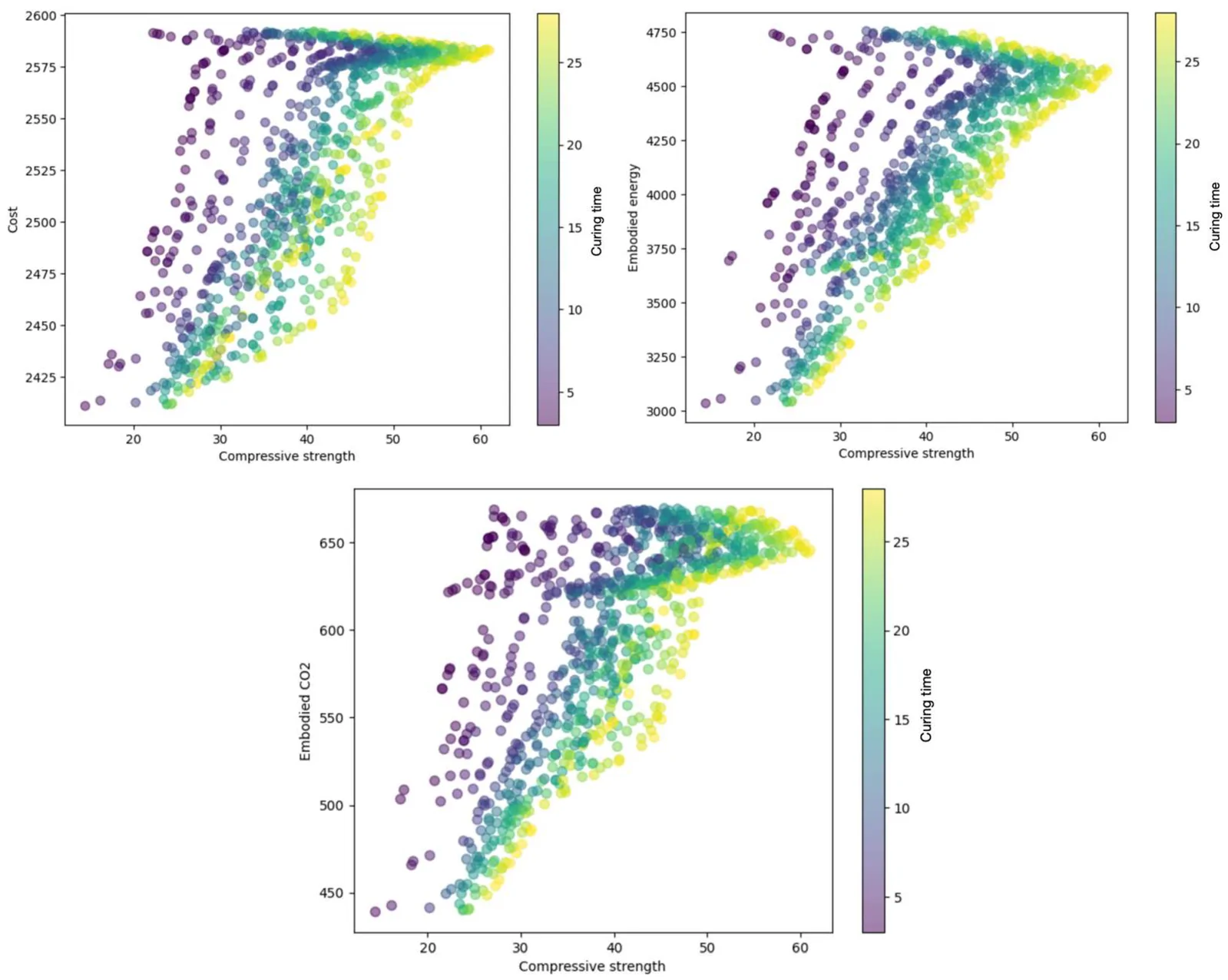
Scatter plots regarding the relationships between compressive strength and cost, energy consumption, and carbon emissions.

Let $x=[x_{1},x_{2},x_{3},x_{4}]$, where $x_{1}$, $x_{2}$, and $x_{3}$ represent the content of CG, SS, and BC, respectively, and $x_{4}$ denotes the curing time. A set of 1000 random samples of x was generated, and scatter plots (see Figure 14) were employed to analyze the relationships between compressive strength and cost, energy consumption, and carbon emissions. The results reveal a clear trade-off between strength and the other three objectives. At any given curing time, higher strength is generally accompanied by higher cost, higher energy consumption, and higher carbon emissions.

##### Solving the Bi-Objective Pareto Front

Given the inherent conflicts between strength and the other objectives, determining the Pareto front is essential. Specific examples are listed below.

(1) Pareto Front for Strength vs. Cost at 28 Days of Curing

The mathematical formulation is established as follows:

Minimize $F\left(x\right)=\left[-f_{1}\left(x\right),f_{2}\left(x\right)\right]$

Subject to:

$$0\leq x_{1},x_{2}\leq 0.4\;0\leq x_{3}\leq 0.04\;x_{4}=28\;x_{1}\times x_{2}=0\;x_{1}\times x_{3}=0\;x_{2}\times x_{3}=0$$

Here, $x=[x_{1},x_{2},x_{3},x_{4}]$, where $x_{1}$, $x_{2}$, and $x_{3}$ represent the content of CG, SS, and BC, respectively, and $x_{4}$ is the curing time. The function $f_{1}$ denotes the strength prediction model, which is based on the three multivariate linear interpolation models developed in Part 1. The function $f_{2}$ represents the cost calculation function.

**Figure 15:**
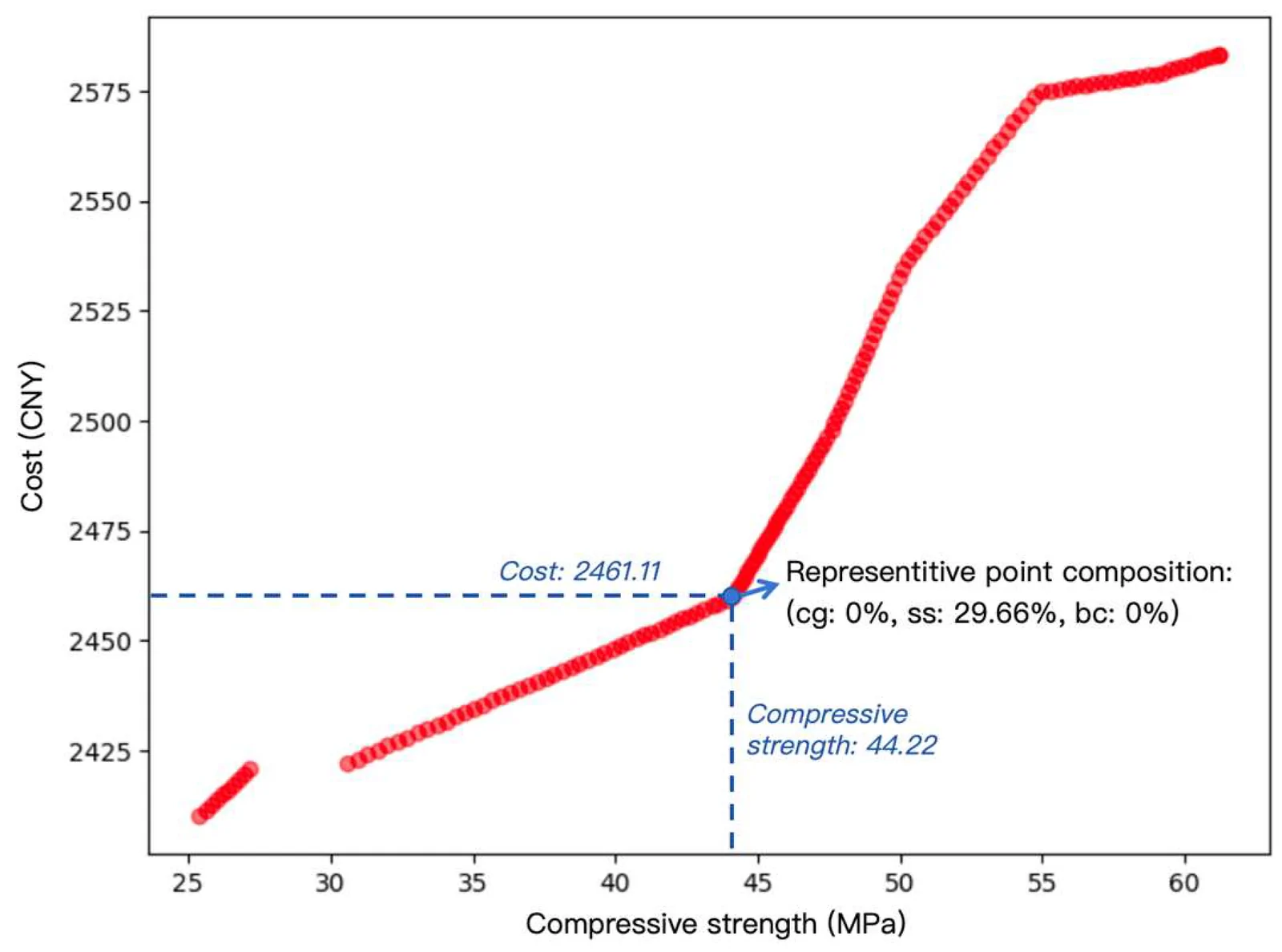
Pareto front based on the NSGA-III algorithm for Example 1.

As shown in Figure 15, the NSGA-III algorithm was employed to solve the optimization problem, with a population size of 150, a maximum of 200 generations, a mutation probability of 0.01, and a crossover probability of 0.9. To improve reproducibility, the optimization was repeated with ten different random seeds. The hypervolume values varied within a narrow range, and the main Pareto trends were stable. The evolutionary solutions were also checked against a dense grid enumeration over the relatively small design space, and the nondominated trends were consistent.

**Figure 16:**
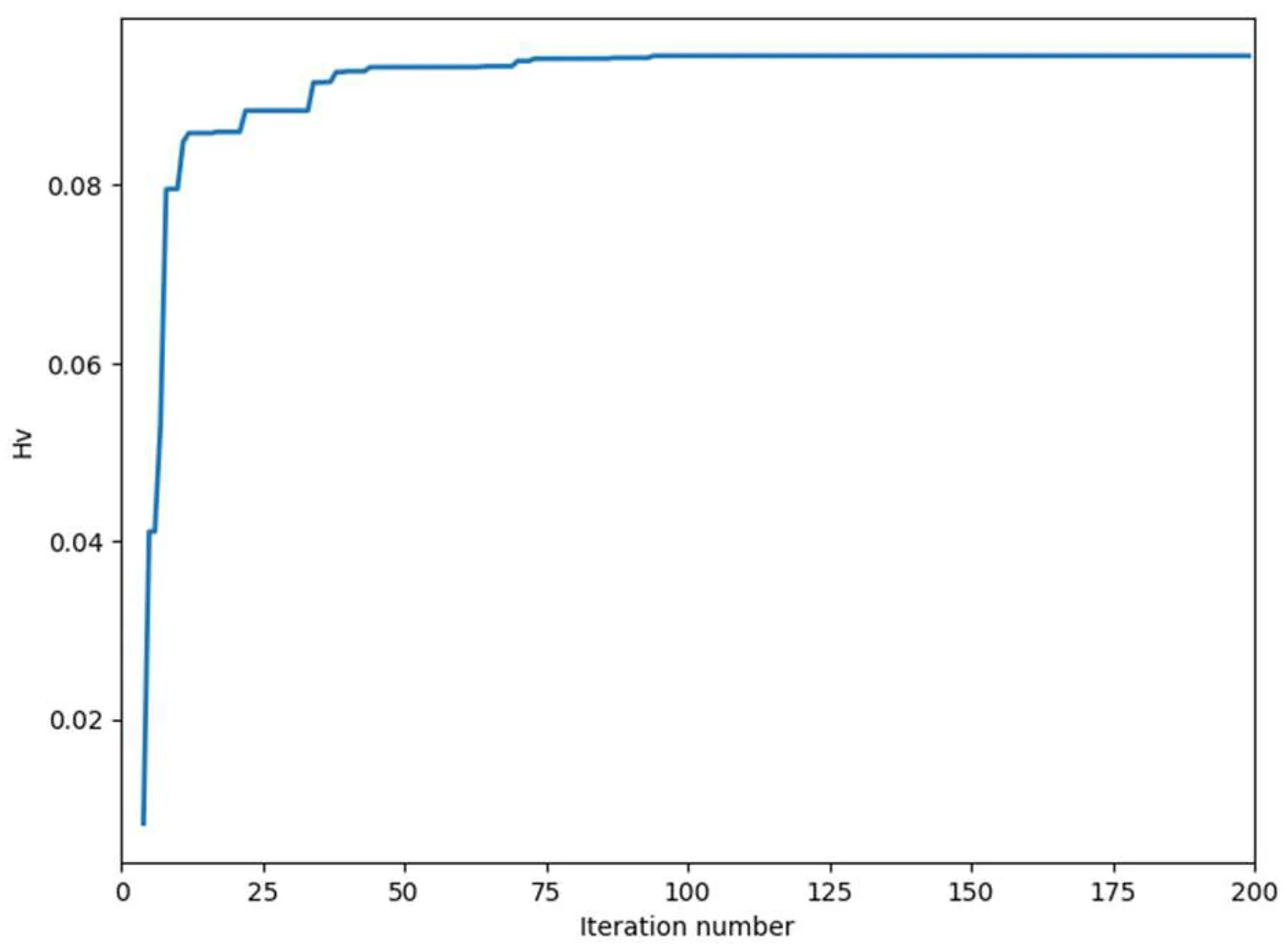
HV indicator progression illustration for Example 1.

The progression of the hypervolume (HV) indicator during the optimization process is shown in Figure 16, demonstrating good convergence of the algorithm.

(2) Pareto Front for Strength vs. Energy Consumption at 28 Days of Curing

The mathematical formulation is established as follows:

$$\mathrm{Minimize}\;F\left(x\right)=\left[-f_{1}\left(x\right),f_{3}\left(x\right)\right]\;\mathrm{Subject}\;\mathrm{to}:\;0\leq x_{1},x_{2}\leq 0.4\;0\leq x_{3}\leq 0.04\;x_{4}=28\;x_{1}\times x_{2}=0\;x_{1}\times x_{3}=0\;x_{2}\times x_{3}=0$$

Here, $f_{1}$ is the same as above and $f_{3}$ represents the energy consumption calculation function (as described in the manuscript; details omitted here).

**Figure 17:**
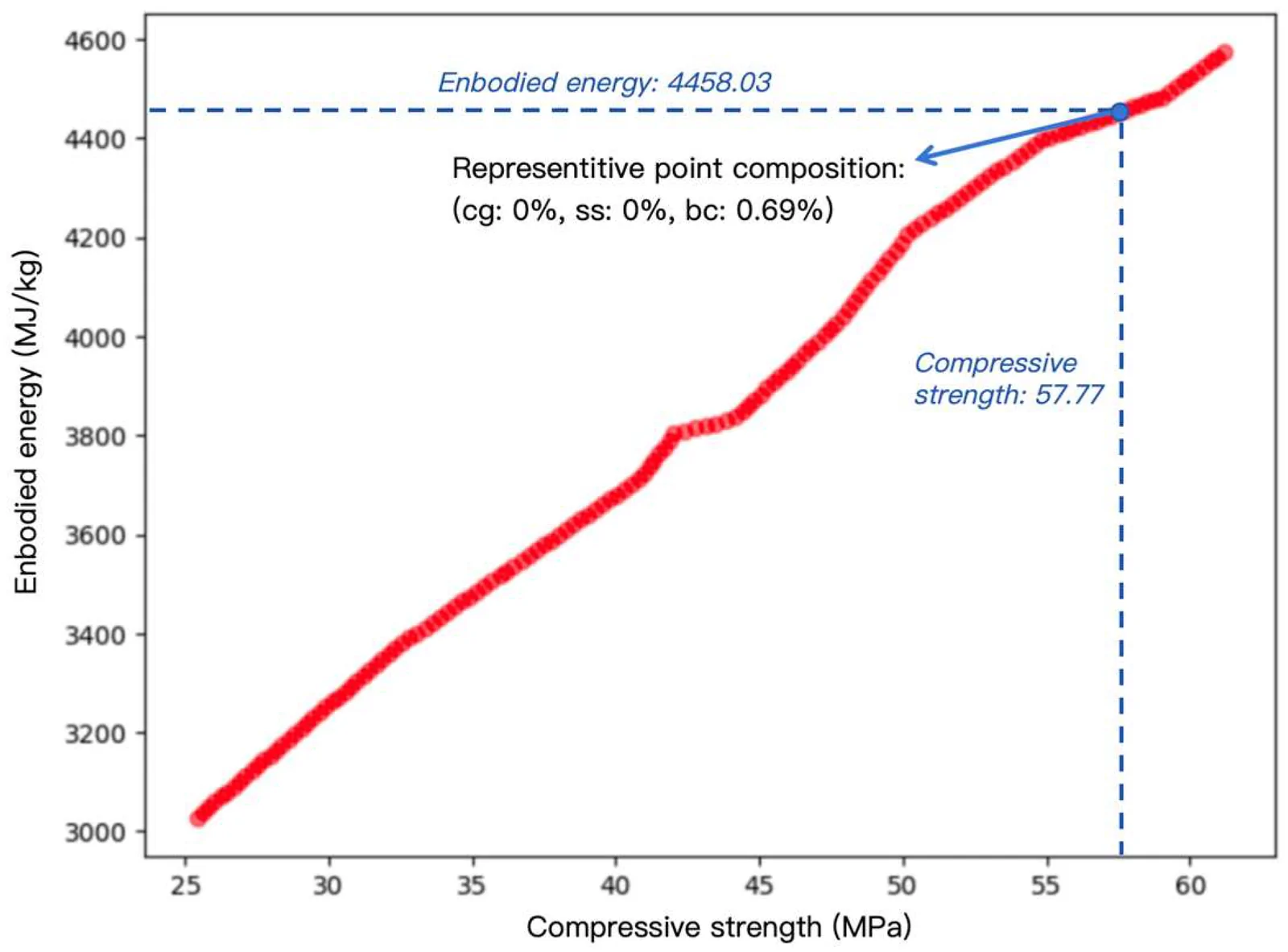
Pareto front based on the NSGA-III algorithm for Example 2.

The NSGA-III algorithm was applied with the same parameter settings as above (population size = 150, maximum generations = 200, mutation probability = 0.01, and crossover probability = 0.9). The obtained Pareto front is shown in Figure 17.

**Figure 18:**
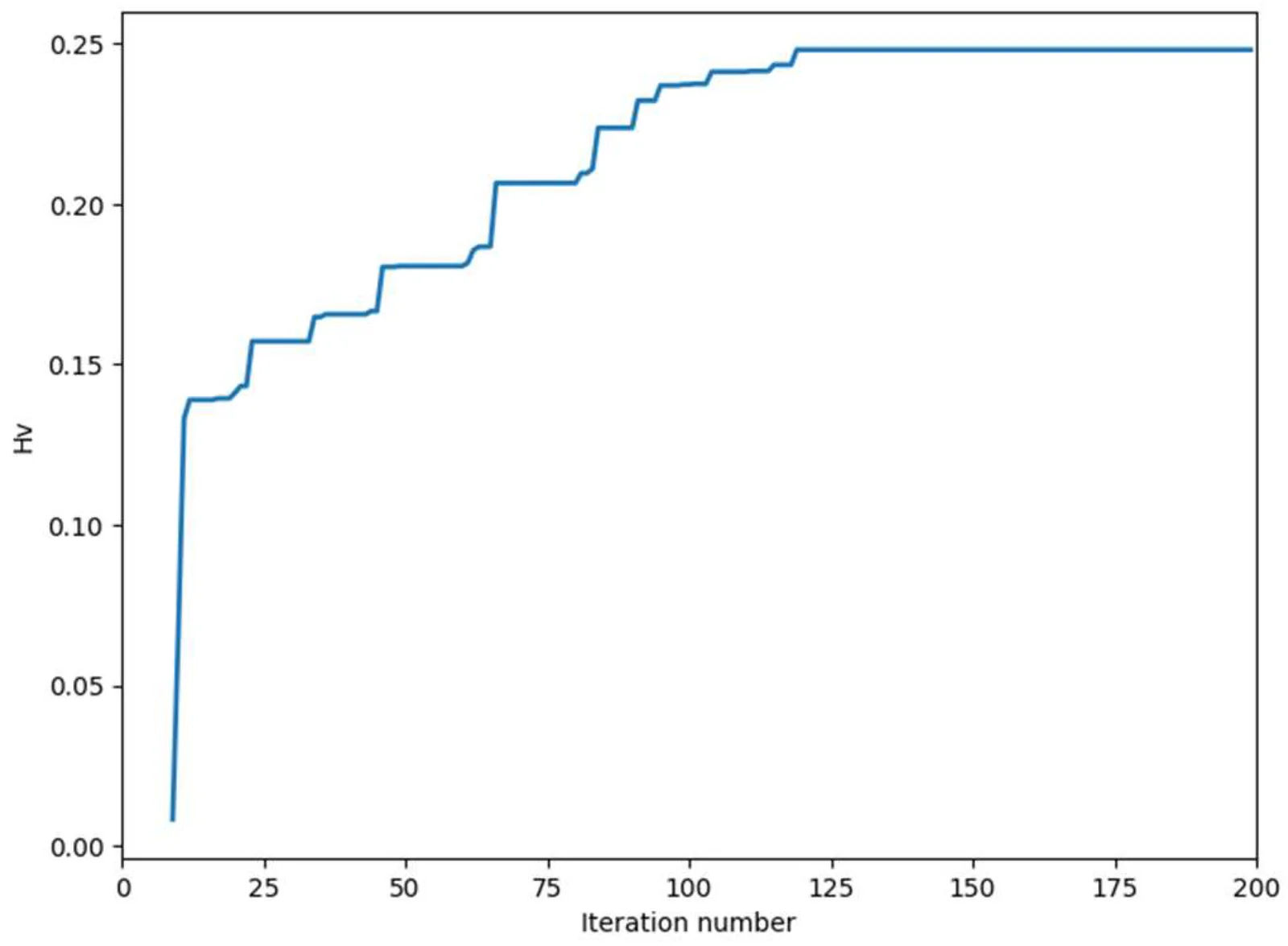
HV indicator progression illustration for Example 2.

The progression of the HV indicator during the optimization process is shown in Figure 18, which also indicates good convergence of the algorithm.

(3) Pareto Front for Strength vs. Carbon Emissions at 28 Days of Curing

The mathematical formulation is established as follows:

$$\mathrm{Minimize}\;F\left(x\right)=\left[-f_{1}\left(x\right),f_{4}\left(x\right)\right]\;\mathrm{Subject}\;\mathrm{to}:\;0\leq x_{1},x_{2}\leq 0.4\;0\leq x_{3}\leq 0.04\;x_{4}=28\;x_{1}\times x_{2}=0\;x_{1}\times x_{3}=0\;x_{2}\times x_{3}=0$$

Here, $f_{1}$ is the same as above and $f_{4}$ represents the carbon emission calculation function (as described in the manuscript; details omitted here).

**Figure 19:**
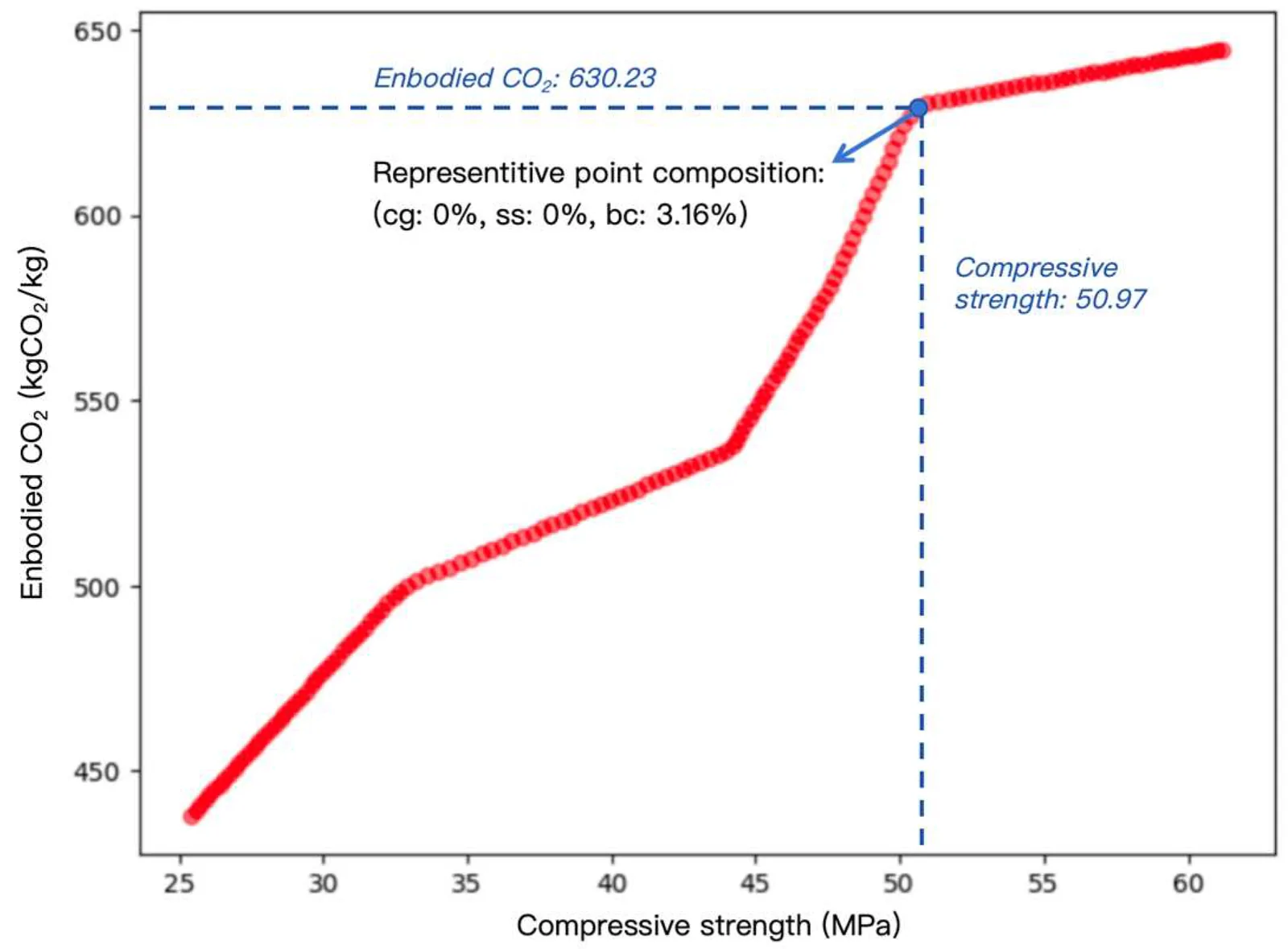
Pareto front based on the NSGA-III algorithm for Example 3.

The NSGA-III algorithm was applied with the same parameter settings as above. The obtained Pareto front is shown in Figure 19.

**Figure 20:**
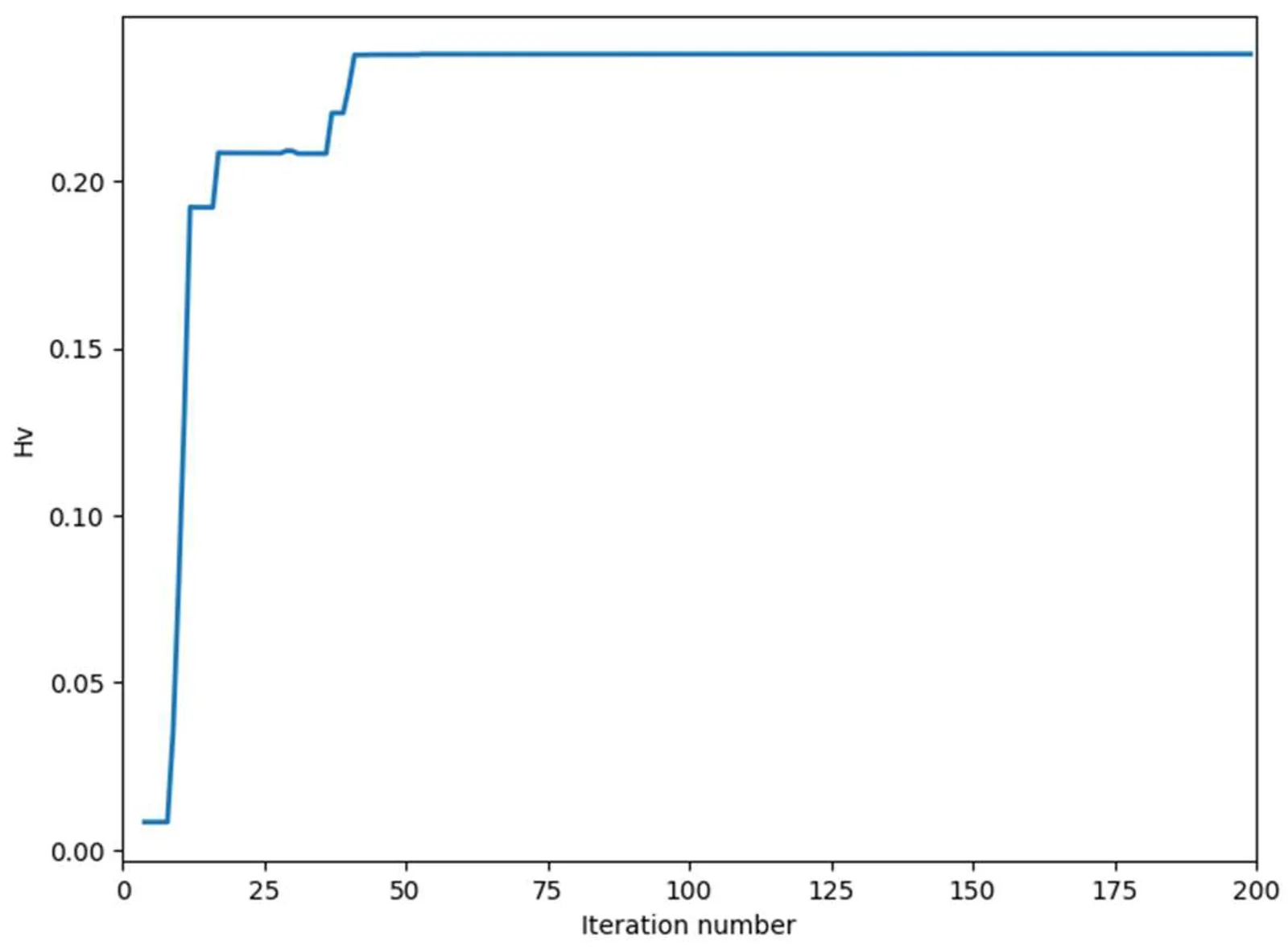
HV indicator progression illustration for Example 3.

The progression of the HV indicator during the optimization process is shown in Figure 20, also demonstrating good convergence of the algorithm.

#### 3.4.3. Four-Objective Optimization

In this section, a more systematic approach was adopted by simultaneously considering all four objectives to solve the four-objective Pareto front. The problem was formulated as follows:

$$\mathrm{Minimize}\;F\left(x\right)=\left[-f_{1}\left(x\right),f_{2}\left(x\right),f_{3}\left(x\right),f_{4}\left(x\right)\right]\;\mathrm{Subject}\;\mathrm{to}:\;0\leq x_{1},x_{2}\leq 0.4\;0\leq x_{3}\leq 0.04\;x_{4}=28\;(\mathrm{Note}:\;\mathrm{Curing}\;\mathrm{time}\;\mathrm{is}\;a\;\mathrm{constant}\;\mathrm{here},\;\mathrm{which}\;\mathrm{means}\;28\;\mathrm{days})\;x_{1}\times x_{2}=0\;x_{1}\times x_{3}=0\;x_{2}\times x_{3}=0$$

Here, the definitions of x, $f_{1}$, $f_{2}$, $f_{3}$, and $f_{4}$ remain consistent with previous sections. The NSGA-III algorithm was employed to solve this problem, with a population size of 300, a maximum of 100 generations, a mutation probability of 0.01, and a crossover probability of 0.9. (Note: These settings differ from previous configurations.) The Pareto optimal solution set was obtained, and the example solutions for the Pareto front with their corresponding objective values are presented in Table 7.

#### 3.4.4. Illustrative Optimization Example

To illustrate the application of the proposed optimization framework, a constrained optimization example is considered for a curing time of 28 days. The objective is to determine an optimized mix proportion that maximizes compressive strength under the constraints of cost < 2500 CNY, energy consumption < 4000 MJ/m^3^, and carbon emissions < 600 kg CO_2_-eq/m^3^. This example is intended only to demonstrate the use of the framework.

The mathematical formulation of this problem is as follows:

$$\mathrm{Maximize}\;f_{1}\left(x\right)\;\mathrm{Subject}\;\mathrm{to}:\;0\leq x_{1},x_{2}\leq 0.4\;0\leq x_{3}\leq 0.04\;x_{4}=28\;(\mathrm{Note}:\;\mathrm{Curing}\;\mathrm{time}\;\mathrm{is}\;28\;\mathrm{days})\;x_{1}\times x_{2}=0\;x_{1}\times x_{3}=0\;x_{2}\times x_{3}=0\;f_{2}(x)<2500\;f_{3}(x)<4000\;f_{4}(x)<600$$

**Figure 21:**
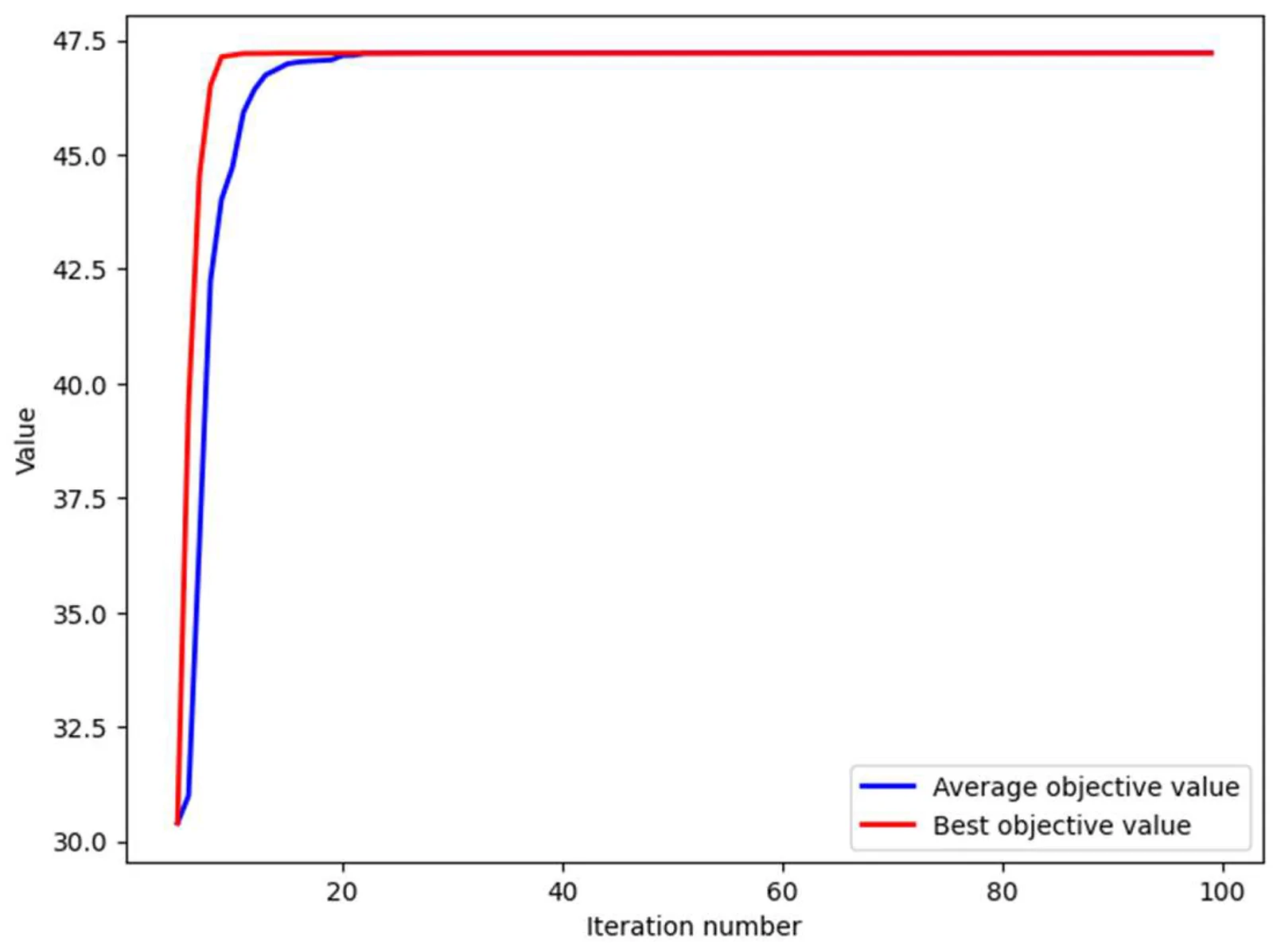
Solution process of DE algorithm.

Here, the definitions of x, $f_{1}$, $f_{2}$, $f_{3}$, and $f_{4}$ remain consistent with previous sections. The Differential Evolution (DE) algorithm was applied to solve this problem, with a population size of 150, a maximum of 100 iterations, a crossover probability of 0.8, and a scaling factor of 0.5. (Note: The algorithm and its settings differ from those used above.) The illustrative optimization example yielded an SS content of 0.2110, corresponding to a predicted compressive strength of 47.21 MPa. The convergence of the DE algorithm for this illustrative example is shown in Figure 21.

## 4. Conclusions

This research systematically investigates the compressive strength of cement mortars modified separately with SS, CG and BC after up to 28 days of curing and establishes a single-SCM multi-objective optimization framework. Experimental results suggest SS as a material with latent hydraulic activity, CG as primarily an inert filler and BC as a performance-enhancing cement replacement within a narrow optimal dosage range. The developed multi-objective optimization framework integrates accurate strength prediction models with advanced algorithms to effectively balance the competing objectives of mechanical strength, economic cost, energy consumption and carbon emissions. An illustrative optimization example is provided to show how the proposed framework can be used to identify mixture proportions that satisfy the specified strength, cost, energy, and carbon constraints. However, the optimized mixture was not experimentally prepared or tested in this study. Therefore, the predicted performance should be regarded as a model-based result and requires future experimental validation. This work provides both fundamental understanding of waste material valorization in cementitious composites and a practical methodology for designing sustainable construction materials, offering valuable insights for advancing green building technologies through strategic utilization of industrial by-products.

It should be noted that the conclusions are limited to short-term compressive strength and preliminary economic and environmental screening of laboratory mortars. This study was intended as a preliminary attempt to establish a test-optimization integrated framework. Therefore, the compressive strength was taken as the main performance index. In particular, the long-term chemical stability of biochar in cementitious systems was not evaluated in this study. Although BC exhibited beneficial effects within the tested curing period (up to 28 days), biochar may undergo physical and chemical degradation over extended service life, which could affect the long-term performance of BC-modified mortars. Therefore, the conclusions regarding BC are limited to the curing ages investigated. Long-term performance of SS, CG and BC in cementitious matrices should be systematically investigated in future work.

## Figures and Tables

**Table 1:** Chemical compositions of cement, SS, CG and BC.

	SiO_2_	CaO	Al_2_O_3_	Fe_2_O_3_	MgO
Cement	22.07	65.3	5.84	3.46	1.72
SS	16.1	40.1	3.72	21.56	7.94
CG	57.94	0.54	26.44	7.68	-
BC	54.32	8.34	12.84	13.12	-

**Table 2:** Mix proportions.

	Water-to-Binder Ratio	Binder-to-Sand Ratio	Waste Material Replacement Ratio (wt%)
SS0-CM	0.35	1:3	0
SS10-CM	0.35	1:3	10
SS20-CM	0.35	1:3	20
SS30-CM	0.35	1:3	30
SS40-CM	0.35	1:3	40
CG0-CM	0.35	1:3	0
CG10-CM	0.35	1:3	10
CG20-CM	0.35	1:3	20
CG30-CM	0.35	1:3	30
CG40-CM	0.35	1:3	40
BC0-CM	0.35	1:3	0
BC1-CM	0.35	1:3	1
BC2-CM	0.35	1:3	2
BC3-CM	0.35	1:3	3
BC4-CM	0.35	1:3	4

Note: SS0-CM, CG0-CM, and BC0-CM refer to the same control mortar. The control specimens were prepared in one common batch and used as the reference for all SCM series.

**Table 3:** Cost, embodied energy and embodied CO_2_ of each component.

Raw Material	Cost (CNY/kg)	Embodied Energy (MJ/kg)	Embodied CO_2_ (kgCO_2_/kg)
Cement	0.708	5.732	0.98
Sand	1	0.341	0.023
Water	0.008	0.019	0.0002
PCE-SP	19	11.18	0.75
SS	0.1	2.75	0.275
CG	0.055	0.3	0.06
BC	1.4	20	−1

**Table 4:** Required mass of each component to produce 1 m^3^ CG-CM.

CG Contents	Cement (kg/m^3^)	Sand (kg/m^3^)	Water (kg/m^3^)	PCE-SP (kg/m^3^)	CG (kg/m^3^)
0	629.3	1887.9	220.3	12.6	0
10%	566.37	1887.9	220.3	12.6	62.93
20%	503.44	1887.9	220.3	12.6	125.86
30%	440.51	1887.9	220.3	12.6	188.79
40%	377.58	1887.9	220.3	12.6	251.72

**Table 5:** Required mass of each component to produce 1 m^3^ SS-CM.

SS Contents	Cement (kg/m^3^)	Sand (kg/m^3^)	Water (kg/m^3^)	PCE-SP (kg/m^3^)	SS (kg/m^3^)
0	629.3	1887.9	220.3	12.6	0
10%	566.37	1887.9	220.3	12.6	62.93
20%	503.44	1887.9	220.3	12.6	125.86
30%	440.51	1887.9	220.3	12.6	188.79
40%	377.58	1887.9	220.3	12.6	251.72

**Table 6:** Required mass of each component to produce 1 m^3^ BC-CM.

BC Contents	Cement (kg/m^3^)	Sand (kg/m^3^)	Water (kg/m^3^)	PCE-SP (kg/m^3^)	BC (kg/m^3^)
0	629.3	1887.9	220.3	12.6	0
1%	623.01	1887.9	220.3	12.6	6.29
2%	616.72	1887.9	220.3	12.6	12.58
3%	610.43	1887.9	220.3	12.6	18.87
4%	604.14	1887.9	220.3	12.6	25.16

**Table 7:** Example solutions and their corresponding values for each objective.

CG Content	SS Content	BC Content	Curing Time	Strength	Price	Energy Consumption	Carbon Emissions
0.2510	0.0000	0.0000	28	36.47	2471.45	3537.85	524.29
0.0000	0.2224	0.0000	28	46.82	2489.52	3978.67	570.97
0.0000	0.0000	0.0136	28	59.86	2580.53	4518.17	652.67

Note. The three representative solutions in Table 7 were selected using an explicit criterion. The first solution represents the lowest-cost region, the second solution represents a balanced SS-based region, and the third solution represents the highest-strength region. Prediction uncertainty is considered by combining the LOOCV MAE with the objective values, so the representative solutions should be understood as robust illustrative choices rather than exact optimal designs. The terms Pareto optimal solution set and Pareto front are equivalent. The Pareto optimal solution set refers to the collection of all solutions that satisfy the Pareto optimality condition in a multi-objective optimization problem. Specifically, for any solution in this set, it is impossible to improve one objective without deteriorating at least one other objective.

## Data Availability

The original contributions presented in the study are included in the article, further inquiries can be directed to the corresponding author.
